# Loss of *Golga7* Suppresses Oncogenic *Nras*‐Driven Leukemogenesis without Detectable Toxicity in Adult Mice

**DOI:** 10.1002/advs.202412208

**Published:** 2025-03-17

**Authors:** Bo Jiao, Lei Yan, Rui Zhang, Wei Huang, Xinru Wang, Chenxuan Liu, Peihong Wang, Pengfei Xu, Jinzeng Wang, Zhou Fang, Donghe Li, Zhizhou Xia, Jiaoyang Li, Shiyu Ji, Qianqian Zhang, Min Wu, Shengyue Wang, Ping Liu, Ruibao Ren

**Affiliations:** ^1^ Shanghai Institute of Hematology State Key Laboratory of Medical Genomics National Research Center for Translational Medicine at Shanghai Ruijin Hospital Shanghai Jiao Tong University School of Medicine Shanghai 200025 China; ^2^ International Center for Aging and Cancer Department of Hematology of The First Affiliated Hospital Hainan Medical University Haikou 571199 China

**Keywords:** Golga7, membrane trafficking, palmitoylation, Ras, therapeutic target

## Abstract

*NRAS* mutations are prevalent in human hematological malignancies and are also common in certain solid tumors, including melanoma and colon cancer. Despite their crucial role in oncogenesis, no effective therapies targeting NRAS have been developed. Inhibiting NRAS localization to the plasma membrane (PM) represents a promising strategy for cancer therapy, as its oncogenic signaling relies on PM localization. Knocking out *Golgin subfamily A member 7 (Golga7)*, an accessory protein of RAS palmitoyltransferases, through a conditional gene editing approach drastically suppresses the development of myeloid leukemia induced by the activation of *Nras^G12D/G12D^* knock‐in alleles in mice. The loss of *Golga7* disrupts NRAS^G12D^ PM localization in bone marrow cells without altering the level of NRAS^G12D^ palmitoylation. Notably, *Golga7* is dispensable for normal hematopoiesis in adult mice. While constitutive *Golga7* knockout leads to embryonic lethality, the ubiquitous knockout of *Golga7* induced in adult mice does not manifest any measurable toxic effects. These findings indicate that GOLGA7 is an effective and safe therapeutic target for *NRAS*‐driven leukemias.

## Graphical abstract

*NRAS* mutations are widespread in hematologic malignancies. Our study shows that GOLGA7 serves as a safe and effective therapeutic target for *NRAS*‐driven leukemia. Loss of *Golga7* in adult mice effectively suppresses *Nras^G12D^*‐driven myeloproliferative neoplasm by disrupting its PM localization and impairing subsequent MAPK signaling, without affecting normal hematopoiesis or causing detectable toxicity, although it leads to embryo lethality.

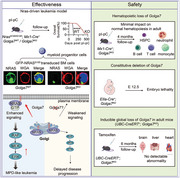

## 1 Introduction

Rat sarcoma (RAS) proteins are small GTPases that act as binary molecular switches in signal transduction pathways regulating cell proliferation, survival, and differentiation.^[^ ^1^ ^]^ There are four homologous RAS proteins in mammals, NRAS, KRAS4A/4B (variants of KRAS), and HRAS, which are encoded by three RAS family genes: *NRAS*, *KRAS*, and *HRAS*, respectively.^[^ ^2^, ^3^ ^]^ Activating *RAS* mutations are found in 20–30% of human cancers.^[^ ^4^, ^5^ ^]^ Particularly in hematologic malignancies, *NRAS* hotspot mutations are the most frequent oncogenic *RAS* isoforms,^[^ ^6^ ^]^ which are widely distributed in various malignant myeloid diseases, including acute myeloid leukemia (ranging from 12 to 21%),^[^ ^7^ ^]^ myelodysplastic syndromes (5%),^[^ ^8^ ^]^ chronic myelomonocytic leukemia (CMML, 15%)^[^ ^9^ ^]^ and juvenile myelomonocytic leukemia (20%).^[^ ^8^, ^10^, ^11^, ^12^ ^]^ In spite of remarkable advancements in utilizing KRAS^G12C^ inhibitors to treat solid tumors in recent years,^[^ ^13^, ^14^, ^15^ ^]^ there remains a paucity of clinically accessible medications for *NRAS*‐driven malignancies.^[^ ^16^ ^]^ Hence, interventions specifically targeting oncogenic NRAS signaling are urgently needed.

Like all other RAS isoforms, NRAS‐dependent signaling requires the proper translocation of RAS proteins to the plasma membrane (PM), predominantly mediated by post‐translational modifications (PTMs) at the carboxyl terminus (C‐terminus).^[^ ^17^ ^]^ Therefore, targeting correct NRAS subcellular localization is considered a rational approach to suppress its oncogenic signaling.^[^ ^18^, ^19^ ^]^ Specifically, NRAS proteins undergo a series of PTMs on the endoplasmic reticulum (ER), acquiring membrane‐anchoring lipid groups through processes such as cysteine prenylation, proteolysis, and carboxy‐methylation at the C‐terminus, which are common to all RAS isoforms.^[^ ^20^ ^]^ Following prenylation at the ER, palmitoylation is another crucial protein lipidation process that takes place at the Golgi apparatus, conferring additional membrane binding affinity to NRAS, as well as HRAS and KRAS4A proteins.^[^ ^21^, ^22^ ^]^ Unlike palmitoylated RAS proteins, KRAS4B, the most abundant and essential RAS isoform, directly traffics to the PM from the ER following prenylation, facilitated by the electrostatic interaction between its positively charged poly‐lysine residues in the hypervariable region (HVR) and the negatively charged component of the inner membrane.^[^ ^23^ ^]^ This discrepancy between RAS isoforms may lead to a safe therapeutical window for interventions targeting NRAS palmitoylation. We and others previously found that blocking the NRAS palmitoylation site by substituting cysteine with serine at 181 (C181S) completely inhibits *NRAS* ^*G12D*^‐induced leukemogenesis.^[^ ^24^, ^25^ ^]^ Thus, targeting NRAS palmitoylation process is a feasible therapeutical strategy.

It is well‐known that palmitoylation of NRAS and HRAS is primarily mediated by the palmitoylacyltransferase (PAT) ZDHHC9 (Zinc finger DHHC domain containing 9) and an accessory protein GOLGA7 (Golgin subfamily A member 7, also known as GCP16), which together form a RAS‐PAT complex at the Golgi. Consistently, we previously found that deletion of *Zdhhc9* can reduce the in vivo level of palmitoylated Ras proteins and delay the onset of *Nras*‐driven leukemia in mice; however, it ultimately fails to prevent disease progress and subsequent mortality.^[^ ^26^ ^]^ This is partially due to the redundancy within the PAT subfamily, wherein at least three ZDHHC9 PAT homologs, including ZDHHC14 and ZDHHC18, have alternative specificity to catalyze the palmitoylation of NRAS or HRAS.^[^ ^27^ ^]^

GOLGA7, acting as a communal partner within the RAS‐PAT complex, has been reported to serve as a stabilizer for members of the ZDHHC9 PAT subfamily by binding with the conserved PATs’ C‐terminal cysteine motif in vitro.^[^ ^28^, ^29^, ^30^ ^]^ Despite being a noncatalytic component in the complex, GOLGA7 was recently found to play key roles in regulating NRAS trafficking to the PM and subsequent oncogenic signaling in leukemia cells.^[^ ^31^, ^32^ ^]^ However, its role in suppressing leukemogenesis in vivo remains uncertain. Furthermore, GOLGA7 is almost ubiquitously expressed throughout the bodies of mammals,^[^ ^33^, ^34^ ^]^ posing a potential challenge for developing interventions to target such a presumably essential gene. In this scenario, it is prudent to verify the safety of GOLGA7 deficiency at the whole‐body level in order to avoid disrupting essential functionalities and causing off‐tumor toxicities in normal tissues.

Herein, we used a *Golga7* conditional knockout (KO) mouse line to assess the effects of *Golga7* on the development and progression of *Nras^G12D^*‐mutant CMML‐like myeloproliferative neoplasm (MPN). We also examined the impact of constitutive *Golga7* knockout on normal development and physiology in mice to ascertain whether GOLGA7 serves as a safe and effective therapeutic target for *NRAS*‐driven leukemias.

## 2 Results

### 2.1 *Golga7* Loss Dramatically Suppresses *Nras* ^*G12D*^‐Induced CMML‐Like MPN

**Figure 1:**
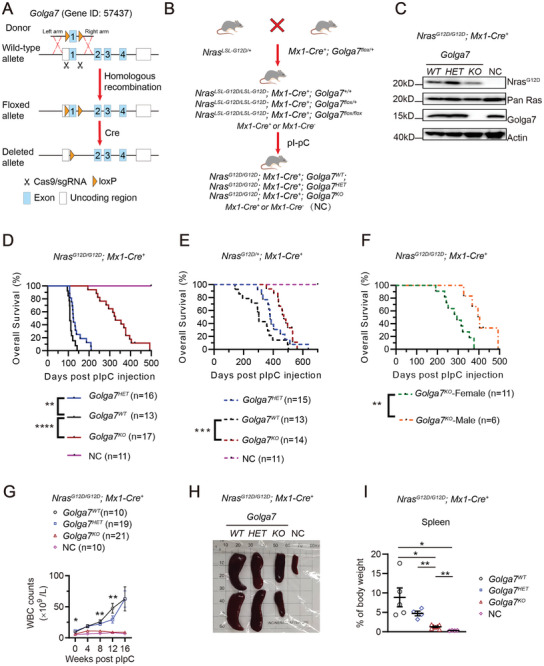
*Golga7* loss suppresses *Nras* ^*G12D*^‐induced CMML‐like MPN. A) The illustration depicts the Golga7 knockout strategy, showing the genetic structures of the wild‐type allele of the murine *Golga7* gene (Gene ID: 57 437, *Mus musculus*), the CRISPR/Cas9 targeting construct, the *loxP*‐floxed allele, and the *Golga7‐*deleted allele following Cre‐mediated recombination. B) Schematic diagram of the procedure to generate the *Nras^G12D^* conditional knock‐in mouse model. We generate three genotypes for investigation: *Nras^G12D/ G12D^; Mx1‐Cre^+^; Golga7^WT^*, *Golga7^HET^*, and *Golga7^KO^*, respectively*. Mx1‐Cre^+^* or *Mx1‐Cre^−^* mice treated with pI‐pC were used as normal control (NC) mice. C) Western blot analysis of protein expression with indicated antibodies to confirm genotypes in peripheral blood (PB) cells. D) Kaplan‐Meier survival curves showing OS of indicated mice within *Nras^G12D/G12D^* genetic background after pI‐pC injection. P‐values were determined using the log‐rank test. E) Kaplan‐Meier survival curves showing OS of indicated mice within *Nras^G12D/+^* genetic background after pI‐pC injection. P‐values were determined using the log‐rank test. F) Kaplan‐Meier survival curves showing sex‐specific OS of mice with *Nras^G12D/G12D^; Mx1‐Cre^+^; Golga7^KO^* genotype after pI‐pC injection. P‐values were determined using the log‐rank test. G) WBC count levels in PB were monitored before and every 4 weeks after pI‐pC injections. *P*‐values were calculated by two‐way ANOVA (*Golga7^WT^* versus *Golga7^KO^*). H) Photograph of spleens from mice of indicated groups. I) The ratio of spleen weight: body weight of indicated mice. *P*‐values were calculated by unpaired Student's *t*‐test. ns, not significant, ^*^ *P*<0.05; ^**^ *P*<0.01; ^***^ *P*<0.001; ^****^ *P*<0.0001.

To evaluate the effect of *Golga7* loss on the development of *Nras^G12D^*‐driven leukemia in vivo, we initially created a conditional *Golga7* (Gene ID: 57 437) gene knockout mouse line based on Cre‐loxP recombination system by employing CRISPR/Cas9 gene editing technology (**Figure** **1**A). We then generated an inducible hematopoietic tissue specific knockout mouse line with distinct genotypes by crossing mice carrying *loxP*‐flanked (floxed) copy of the gene (*Golga7^flox/+^*) and the Mx1‐Cre allele (*Mx1‐Cre^+^*) with a mouse line harboring endogenous *Nras^LSL‐G12D/+^*. After activation of Cre recombinase under the control of the *Myxovirus resistance 1* (*Mx1*) promoter by injecting 2 doses of polyinosinic‐polycytidylic acid (pI‐pC, 250 µg per dose), we successfully obtained mice with three distinct genotypes, e.g. *Nras^G12D/G12D^; Mx1‐Cre^+^; Golga7^WT^*, *Golga7^HET^* and *Golga7^KO^*, respectively (Figure 1B). Also, *Mx1‐Cre^+^* or *Mx1‐Cre^−^* mice were used as normal control (NC).^[^ ^35^ ^]^ Genotyping analysis confirmed the deletion of floxed alleles by genomic polymerase chain reaction (PCR) (Figure S1A, Supporting information). After *Golga7* deletion and activation of oncogenic *Nras^G12D^* using pI‐pC, the endogenous expression of Nras^G12D^ and Golga7 proteins was confirmed by western blot analysis using peripheral blood (PB) cells from mice with different genotypes (Figure 1C).

During a 500‐day observation period, *Nras^G12D/G12D^; Mx1‐Cre^+^; Golga7^KO^* mice showed a dramatically extended overall survival (OS) time compared with the *Golga7^WT^* counterparts (median, 327 days vs 107 days, respectively; *P*<0.0001) (Figure 1D). Also, *Nras^G12D/G12D^; Mx1‐Cre^+^; Golga7^HET^* mice had prolonged OS (median, 124 days; *P* = 0.0061) when compared to *Golga7^WT^* counterparts, suggesting a moderate dose‐dependent inhibitory effect of *Golga7* loss on *Nras* ^*G12D*^‐driven leukemia development (Figure 1D). Similarly, in the mouse cohort of heterozygotic *Nras^G12D/+^* (Figure 1E), mice bearing *Golga7^KO^* and Golga7*^HET^* also showed a gradually extended OS compared to the *Golga7^WT^* animals in a gene dose‐dependent manner (median, 379 days and 471 days vs 303.5 days, respectively). Notably, we observed sex‐specific differences in OS existed within the cohort of *Nras^G12D/G12D^; Mx1‐Cre^+^; Golga7^KO^* animals, with males exhibiting relatively longer survival time compared to females (median, 400 days vs 294 days, respectively; *P* = 0.0036) (Figure 1F).

It is well documented that *Nras^G12D/G12D^; Mx1‐Cre^+^* mice exhibit progressive CMML‐like MPN in blood after pI‐pC induction.^[^ ^26^, ^35^, ^36^ ^]^ Therefore, we monitored PB samples by conducting complete blood count (CBC) analyses every four weeks for 16 weeks post pI‐pC treatment. The results indicated that white blood cell (WBC) counts were reduced in *Nras^G12D/G12D^; Mx1‐Cre^+^; Golga7^KO^* mice compared to *Golga7^WT^* and *Golga7^HET^* mice (Figure 1G). Using flow cytometry to analyze the cellular composition of PB, we found that *Golga7^KO^* mice exhibited only a moderate increase in Mac1^+^ cells compared to NC mice throughout the 16‐week observation period (Figure S1B, Supporting information).

To elucidate differences in hematopoietic phenotype among the different genotypes during leukemia progress, mice were euthanized 16 weeks after pI‐pC injections. *Nras^G12D/G12D^; Mx1‐Cre^+^; Golga7^KO^* mice displayed significantly smaller spleen sizes when compared to *Golga7^WT^* or *Golga7^HET^* counterparts (Figure 1H,I). Histological analysis of the spleen and liver revealed leukemia infiltration in *Golga7^WT^* and *Golga7^HET^* mice but not in *Golga7^KO^* or NC mice (Figure S1C, Supporting information). More importantly, flow cytometry analysis of spleen and bone marrow (BM) cells showed reduced infiltration of Mac1^+^ myeloid cells in *Golga7^KO^* compared to *Golga7^WT^* and *Golga7^HET^* (Figure S1D,E, Supporting information).

We further assessed the impact of *Golga7* loss on the hematopoietic stem and progenitor cell (HSPC) compartment by Fluorescence activated Cell Sorting (FACS) (Figure S1F, Supporting information). In line with the previous report,^[^ ^35^ ^]^ BM from *Nras^G12D/G12D^; Mx1‐Cre^+^; Golga7^WT^* mice displayed an expanded Lin^−^Kit^+^ (LK) cell population as well as common myeloid progenitor (CMP) (LK/CD16/32^−^CD34^+^), representing a typical committed myeloid leukemia phenotype. Importantly, *Nras^G12D/G12D^; Mx1‐Cre^+^; Golga7^KO^* mice showed a significantly decreased population of CMP compared with *Golga7^WT^* mice (P<0.01), but still higher than that in NC mice (P<0.01). In contrast, no significant differences among genotypes were found in granulocyte‐monocyte progenitor (GMP) (LK/CD16/CD32^+^) or megakaryocyte‐erythroid progenitor (MEP) (LK/CD16/CD32^−^CD34^−^) among groups (Figure S1G, Supporting information). Also, *Nras^G12D/G12D^; Mx1‐Cre^+^; Golga7^WT^* mice exhibited significantly reduced long‐term hematopoietic stem cells (LT‐HSCs; LSK/CD135^−^ CD150^+^CD48^−^) and short‐term hematopoietic stem cells (ST‐HSC; LSK/CD135^−^CD150^−^CD48^−^ compared to NC, which was partially ameliorated in *Golga7^KO^* mice (Figure S1H, Supporting information). Altogether, these data suggest that *Golga7* loss mainly constrains *Nras* ^*G12D*^‐induced leukemic cell expansion in CMP and preserves normal hematopoiesis in HSPCs to some extent.

To further compare the leukemogenic potential of BM cells among different genotypes, we performed colony‐forming unit (CFU) assays in semi‐solid medium with or without cytokine stimulation. Consistent with previous reports,^[^ ^26^, ^37^ ^]^ BM cells from *Nras^G12D/G12D^; Mx1‐Cre^+^; Golga7^WT^* and *Golga7^HET^* mice formed considerable colonies in M3231 medium in the presence or absence of murine granulocyte‐macrophage colony stimulating factor (mGM‐CSF) In contrast, BM cells from *Golga7^KO^* mice formed far fewer colonies, regardless of cytokine presence (Figure S1I, Supporting information).

Collectively, these data demonstrate that the loss of *Golga7* significantly suppresses oncogenic *Nras*‐driven leukemogenesis in vivo.

### 2.2 Loss of *Golga7* Inhibits PM Localization of GFP‐NRAS^G12D^ and Diminishes RAS Signaling in Murine BM Cells

**Figure 2:**
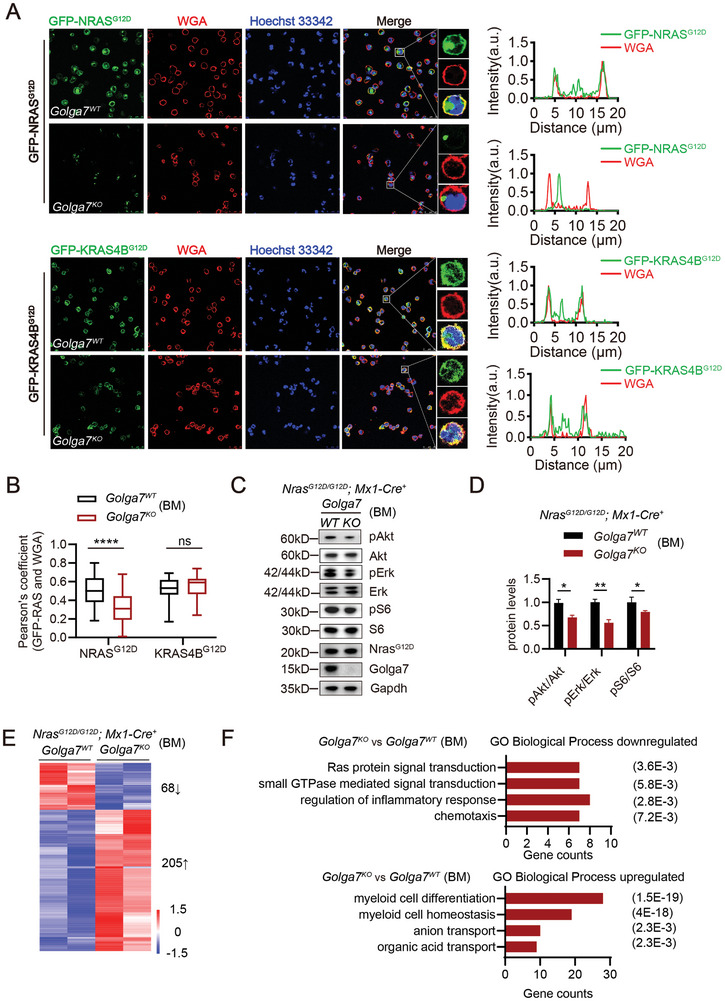
*Golga7* loss disrupts PM localization of GFP‐NRAS^G12D^ and inhibits *Nras^G12D^*‐induced signaling in BM cells. A) Representative IF confocal images of GFP‐NRAS^G12D^ or GFP‐KRAS4B^G12D^‐expressing (green) BM cells from G*olga7^WT^* and *Golga7^KO^* mice with the PM marker wheat germ agglutinin (WGA; red) and nuclear staining (Hoechst 33 342; blue). The original magnification was ×630. Scale bar: 25 µm. B)Pearson's coefficient of GFP and WGA signals (mean ± SEM) of data (A). Values are the mean ± SEM from *n* = 60 cells per group. Statistical comparisons were performed by unpaired Student's *t*‐test in Prism. GFP‐NRAS^G12D^, ^****^ *P*<0.0001; GFP‐KRAS4B^G12D^, ns *P* = 0.1769. C) Whole BM cells from *Nras^G12D/G12D^; Mx1‐Cre^+^; Golga7^WT^* and *Nras^G12D/G12D^; Mx1‐Cre^+^; Golga7^KO^* mice were starved, and their lysates were subsequently subjected to western blot analysis to measure pAkt, pErk, and pS6 levels. D) Quantification of data is shown in (C). Data are the mean ± SEM from three independent experiments. Statistical comparisons were performed by unpaired Student's *t*‐test in Prism. ^*^ *P*<0.05; ^**^ *P*<0.01; ^****^ *P*<0.0001. E) Heatmap of DEGs in *Nras^G12D/G12D^; Mx1‐Cre^+^; Golga7^KO^* cells compared with *Nras^G12D/G12D^; Mx1‐Cre^+^; Golga7^WT^* cells (fold change >1.5 and false discovery rate (FDR) <0.05). F) Gene Ontology (GO) analysis of DEGs in *Nras^G12D/G12D^; Mx1‐Cre^+^; Golga7^KO^* cells. The representative biological processes are shown with numbers of genes in each category and corresponding FDR in parentheses.

A previous study showed that GOLGA7 is uniquely required for the localization of NRAS^G12D^ on the plasma membrane (PM) in human HeLa cells.^[^ ^31^ ^]^ To evaluate this in primary hematopoietic cells, we euthanized pI‐pC‐induced mice *Mx1‐Cre^+^; Golga7^WT^*, and *Golga7^KO^* and infected their BM cells with retroviruses carrying human GFP‐NRAS^G12D^ or GFP‐KRAS4B^G12D^. Immunofluorescence (IF) analysis showed the expected strong PM association of GFP‐NRAS^G12D^ and GFP‐KRAS4B^G12D^ in *Golga7^WT^* BM cells (**Figure** **2**A,B). In contrast, PM localization of GFP‐NRAS^G12D^ was dramatically reduced in *Golga7^KO^* BM cells, whereas localization of GFP‐KRAS4B^G12D^ was unaffected (Figure 2A,B), consistent with the previous report.^[^ ^31^ ^]^

Furthermore, we performed a subcellular fractionation of bone marrow cells from *Nras^G12D/G12D^; Mx1‐Cre^+^; Golga7^WT^* and *Golga7^KO^* mice and detected the level of Nras^G12D^ proteins by western blot (Figure S2A,B, Supporting information). We confirmed that Nras mutant proteins were only significantly reduced in the membrane portion of *Golga7^KO^* cells but not in the cytosolic part nor whole cell lysate, which is consistent to the result from the immunofluorescence assay in Figure 2A. To further confirm this result, we next conducted a classic chase assay to test the protein stability of GFP‐NRAS^G12D^ proteins in GOLGA7^KO^ HeLa cells (Figure S2C,D, Supporting information). By using the cycloheximide to inhibit de novo protein synthesis, we found the levels of total GFP‐NRAS^G12D^ proteins were stable regardless of the presence or absence of GOLGA7 during the 16 h chase. Interestingly, we also noted that GOLGA7 protein was not as stable as NRAS, suggesting the turnover rate of GOLGA7 is much more rapid than NRAS (Figure S2C,D, Supporting information).

Oncogenic NRAS is known to mediate the activation of signaling pathways, including the mitogen‐activated protein kinase (MAPK) pathway, the RAF/MEK/ERK pathway, and the PI3K/AKT/mTOR survival pathway.^[^ ^1^, ^38^, ^39^, ^40^ ^]^ We assumed that Golga7 might affect these pathways through the maturation process of Nras proteins at the level of post‐translational modifications. To detect the effect of Golga7 loss on the signaling as early as possible, we sacrificed the *Nras^G12D/G12D^; Mx1‐Cre^+^; Golga7^WT^* and *Golga7^KO^* mice at 8 weeks after pI‐pC injection. First, our results showed that even at 8 weeks after pI‐pC injection, the *Golga7^WT^* mice had already developed a relatively obvious CMML‐like MPN disease phenotype (Figure S2E–J, Supporting information), while the disease phenotype of the *Golga7^KO^* mice was significantly weaker than that of the WT counterparts. However, when compared with the NC mice, the *Golga7^KO^* mice also already showed a certain phenotype of abnormal myeloid proliferation.

Subsequently, we directly evaluated the phosphorylation level of several MAPK and PI3K/AKT proteins in whole BM cells from indicated genotypes in Figure 2C and found that *Golga7* loss reduced phospho‐Akt, phospho‐Erk, and phospho‐S6 levels in BM cells (Figure 2C,D).

Meanwhile, we also performed RNA sequencing (RNA‐seq) analysis on whole BM cells derived from the cohorts as shown in Figure S2K (Supporting information). We identified 284 common upregulated genes in both the WT and KO compared to NC mice. The Gene Ontology (GO) analysis of the genes was mainly enriched in gene signatures that are relatively classic for abnormal regulation by NRAS mutations, such as inflammatory response, leukocyte migration, and the ERK1/2 cascade pathway (Figure S2K,L, Supporting information).

This indicates that even the *Golga7^KO^* mice already had a significant activation of the abnormal RAS signaling at this stage. Moreover, when further comparing the differentially expressed genes between the WT and KO mice, specifically, a mere total of 273 differentially expressed genes (DEGs) (fold change >1.5 and false discovery rate (FDR) <0.05) in *Nras^G12D/G12D^; Mx1‐Cre^+^; Golga7^KO^* versus *Golga7^WT^*, including 68 downregulated genes and 205 upregulated genes were identified (Figure 2E). These results suggest that loss of *Golga7* diminishes *Nras* ^*G12D*^‐mediated signaling pathways for cell growth and proliferation, and therefore slows the progression of *Nras* ^*G12D*^‐driven CMML‐like MPN(Figure 2F).

### 2.3 *Nras^G12D/G12D^; Mx1‐Cre^+^; Golga7^KO^* Mice Develop CMML‐like MPN After a Prolonged Latency

**Figure 3:**
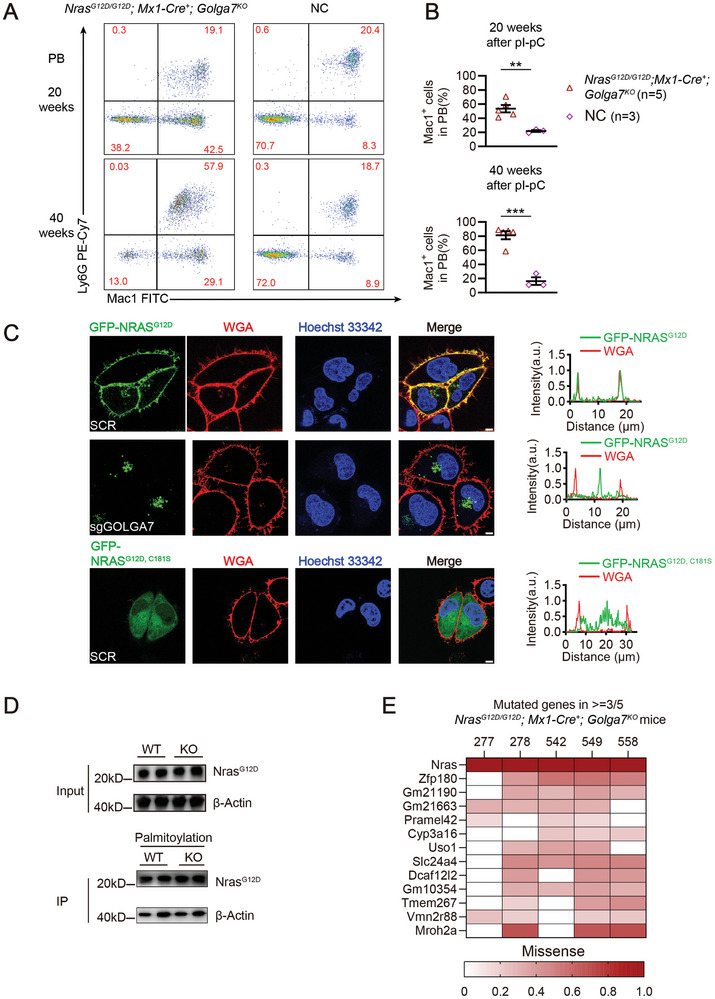
*Nras^G12D/G12D^; Golga7^KO^* mice develop CMML‐like MPN after a prolonged latency. A) Representative flow cytometry analysis of peripheral blood cells from *Nras^G12D/ G12D^; Mx1‐Cre^+^; Golga7^KO^* and NC mice (20 and 40 weeks after pI‐pC injections). B) Quantification of data is shown in (A). Data are the mean ± SEM. Statistical comparisons were performed by unpaired Student's *t*‐test in Prism. ^**^ *P*<0.01; ^***^ *P*<0.001. C) HeLa cells were transfected with scramble single guide RNA (sgRNA, SCR) or sgRNA against GOLGA7 (sgGOLGA7), respectively. Cells with or without sgRNA‐mediated knockout of GOLGA7 were stably expressed GFP‐NRAS^G12D^ via lentiviral infection. A line was drawn across confocal images of cells and the signals for GFP (green), wheat germ agglutinin (WGA; red), and nuclei (Hoechst33342; blue) along the line are plotted. The original magnification was ×630. Scale bar: 5 µm. D) Palmitoylation levels of Nras proteins in spleen cells from *Nras^G12D/ G12D^; Mx1‐Cre^+^; Golga7^WT^* and *Golga7^KO^* mice determined by Acyl‐RAC assay. E) Genomic DNA was extracted from BM cells and spleen of moribund *Nras^G12D/ G12D^; Mx1‐Cre^+^; Golga7^KO^* (*n* = 5) and matched NC mice (*n* = 3) for whole exome sequencing. Recurrent mutations (mutated in ≥3/5 mice) and their variant allele frequencies are shown.

Although the survival time of *Nras^G12D/G12D^; Mx1‐Cre^+^; Golga7^KO^* mice were significantly prolonged compared to *Golga7^WT^* mice, they inevitably developed CMML‐like MPN starting from 20 weeks post pI‐pC, as the Mac‐1^+^ myeloid population gradually elevated in PB (**Figure** **3**A,B). Like *Golga7^WT^* mice, moribund *Nras^G12D/G12D^; Mx1‐Cre^+^; Golga7^KO^* mice displayed CMML‐like MPN phenotypes, with increased granulocytes and monocytes in BM and spleen (Figure S3A, Supporting information).

As previously reported, palmitoylation is an essential process for NRAS^G12D^ leukemogenesis, the C181S mutation inhibited the palmitoylation of oncogenic NRAS and prevented the progression of leukemia.^[^ ^24^, ^25^ ^]^ Since BM cells are relatively small, making it difficult to distinguish subcellular localization, we transduced HeLa cells with lentiviruses expressing human GFP‐NRAS^G12D^. Notably, we found that the localization of GFP‐NRAS^G12D^ in *GOLGA7‐*loss HeLa cells differs from that of palmitoylation‐deficient NRAS^G12D,C181S^. NRAS^G12D^ in *GOLGA7‐*deficient cells is primarily distributed in the perinuclear region and Golgi apparatus, NRAS^G12D,C181S^ is dispersed throughout the cytoplasm in control cells (Figure 3C). As previously described, *GOLGA7* loss does not significantly inhibit the palmitoylation level of NRAS in human cell lines.^[^ ^27^, ^41^ ^]^

To further investigate this in vivo, we used the acyl‐Resin‐Assisted Capture (Acyl‐RAC) method to measure S‐palmitoylation levels of endogenous Nras^G12D^ in spleen cells from *Golga7^WT^* and *Golga7^KO^* mice. Consistently, we did not observe a noticeable decrease in palmitoylated Nras^G12D^ in *Golga7^KO^* spleen cells compared to *Golga7^WT^* cells (Figure 3D), indicating that the altered subcellular localization of NRAS^G12D^ is not due to the decrease in palmitoylation levels. This suggests that GOLGA7 may play an indispensable role in the post‐palmitoylation transport of NRAS from the Golgi to the PM.

To investigate whether the *Nras^G12D/G12D^; Mx1‐Cre^+^; Golga7^KO^* mice acquired additional oncogenic mutations during leukemia development, we performed whole exome sequencing (WES) analysis on BM cells and spleen cells from 5 independent moribund *Golga7^KO^* samples and 3 independent NC samples (Figure S3B, Supporting information). Unexpectedly, we did not detect any well‐known oncogenic mutations (Figure 3E), indicating that Nras may signal from the Golgi endomembrane to sustain the oncogenesis as previously reported.^[^ ^42^, ^43^ ^]^ Interestingly, among the mutated genes detected in moribund *Golga7^KO^* samples, several are potentially involved in intracellular transport. For example, *Uso1* is essential for vesicle transport, particularly between the endoplasmic reticulum and Golgi,^[^ ^44^ ^]^ and *Slc23a4* is involved in ion transport.^[^ ^45^ ^]^ These findings suggest that GOLGA7 is highly likely to be involved in the membrane trafficking process of NRAS post‐palmitoylation. Thus, although *Golga7* loss drastically delays the onset of *Nras* ^*G12D*^‐induced CMML‐like MPN, it is unable to prevent the disease eventually.

### 2.4 Loss of *Golga7* has Minimal Impact on Normal Hematopoiesis in Adult Mice

**Figure 4:**
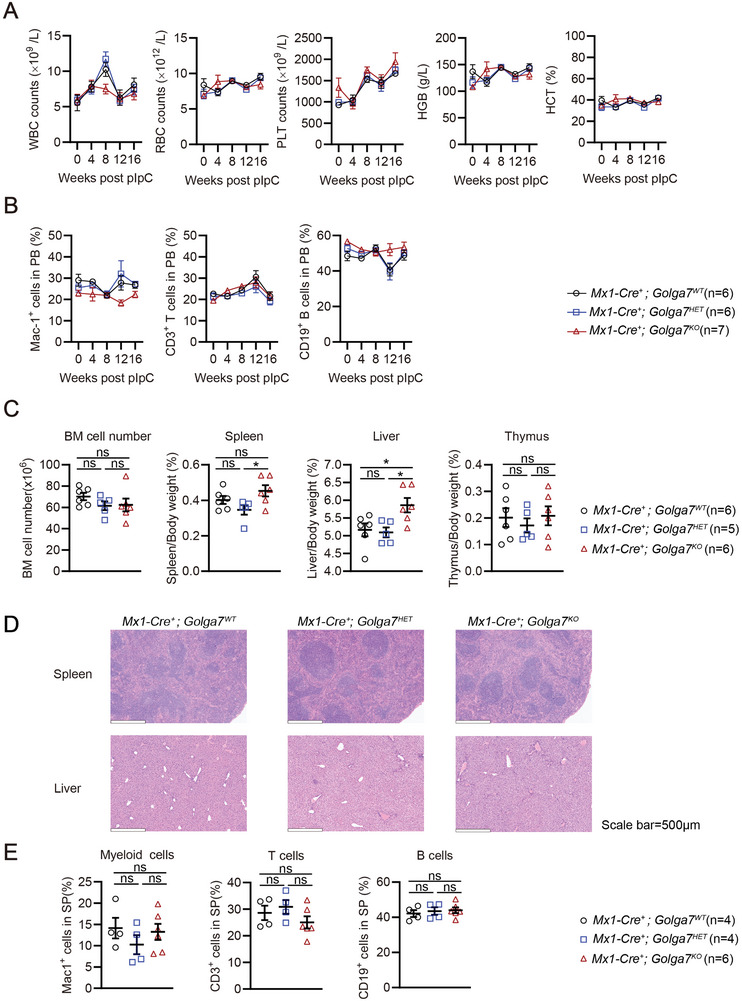
*Golga7* loss has minimal impact on normal hematopoiesis in adult mice. A) The WBC counts, RBC counts, hemoglobin levels, hematocrit levels, and PLT counts from PB were monitored at baseline and every 4 weeks after pI‐pC injections for 16 weeks in indicated mice. Statistical comparisons were performed by unpaired Student's *t*‐test in Prism. B) The frequency of differentiated cells in PB was monitored at baseline and every 4 weeks for 16 weeks after pI‐pC injections. Statistical comparisons were performed by unpaired Student's *t*‐test in Prism. C) The BM cell counts, ratios of spleen weight, liver weight, and thymus weight to body weight were determined in *Mx1‐Cre^+^*; *Golga7^WT^*, *Golga7^HET^*, and *Golga7^KO^* mice, respectively. Data are presented as the mean ±S.D. ^*^ *P*<0.05. Statistical comparisons were performed by unpaired Student's *t*‐test in Prism. D) Representative Hematoxylin and Eosin (H&E) staining of spleen and liver sections at 16 weeks after pI‐pC injections. Scale bar: 500 µm. E) Analysis of the frequencies of differentiated cells from *Mx1‐Cre^+^; Golga7^WT^*, *Golga7^HET^*, and *Golga7^KO^* mice, respectively. Data are presented as mean ± SEM. Statistical comparisons were performed by unpaired Student's *t*‐test in Prism. ns, not significant, ^*^ *P* < 0.05.

Next, to determine whether Golga7 serves as a suitable target for developing targeted therapies, we then assessed its loss in normal hematopoiesis. The PB samples from mice with *Mx1‐Cre^+^; Golga7^KO^* were monitored every 4 weeks for 16 weeks after pI‐pC injection. Throughout this period, *Golga7^KO^* mice maintained normal blood counts (**Figure** **4**A,B). Post‐mortem analysis at 16 weeks after pI‐pC injections showed that BM cell counts and thymus weights were unaffected by *Golga7* loss (Figure 4C). Spleen: body weight ratio was significantly higher in *Golga7^KO^* compared to *Golga7^HET^* animals, and liver: body weight ratio was statistically higher in *Golga7^KO^* compared to both *Golga7^WT^* and *Golga7^HET^* mice (Figure 4C); however, histopathological analysis revealed no significant structural differences in liver and spleen between *Golga7^WT^* and *Golga7^KO^* mice (Figure 4D). The levels of myeloid cells, B cells, and T cells in the spleen were comparable among all genotypes of mice (Figure 4E). We also found *Mx1‐Cre^+^; Golga7^KO^* mice had unaffected HSPCs, despite a slight increase in LT‐HSCs (Figure S4A, Supporting information). CFU assays showed that BM cells from all three groups formed multilineage colonies of granulocytes, erythrocytes, monocytes, and macrophages (GEMM colonies) without significant differences in colony numbers, indicating that *Golga7* loss does not affect the differentiation and proliferation abilities of HSPC (Figure S4B,C, Supporting information). Thus, these results suggest that *Golga7* loss has minimal impact on normal hematopoiesis in adults.

### 2.5 *Golga7* is Essential for Embryo Development

**Figure 5:**
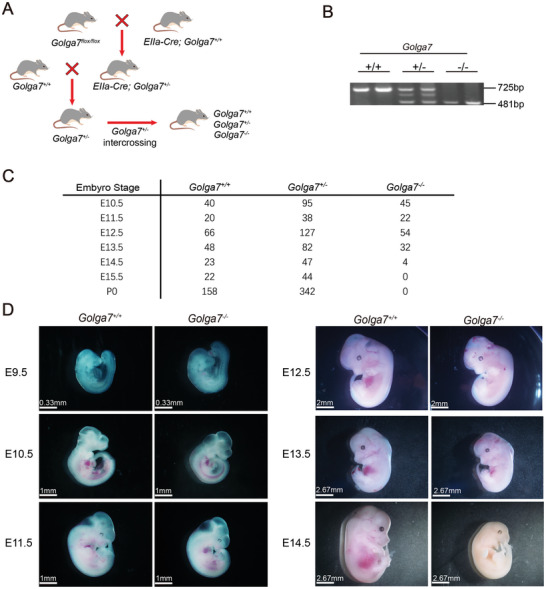
Constitutive loss of *Golga7* leads to embryonic lethality. A) Schematic diagram of the procedure to generate the *Golga7* null mouse model. B) Representative PCR analysis of genomic DNA to confirm genotypes of *Golga7^+/+^, Golga7^+/−^*, and *Golga7^−/‐^* mice. C) Frequency of progeny of indicated genotypes at different stages of development from E10.5 to P0. D) Representative images showing indicated embryos at different stages of development from E9.5 to E14.5.

According to some online gene expression databases (e.g., datasets from Human Protein Atlas and Genotype‐Tissue Expression), GOLGA7 is expressed in various tissues throughout the human body, implying that therapeutic targeting of GOLGA7 may potentially affect multiple biological processes. As previous reports have shown,^[^ ^46^ ^]^ the challenge for therapeutically useful targets lies in maintaining efficacy and safety. To evaluate the safety of GOLGA7 as a potential drug target, we generated a germline Golga7‐null (*Golga7^−/−^*) mouse line. Heterozygous mice carrying the *Golga7^flox/+^* with mice carrying *EIIa‐Cre* were intercrossed as shown in **Figure** **5**A. We further performed genotyping analysis by genomic PCR (Figure 5B). Notably, *Golga7^−/‐^* was not found at weaning and died at mid‐to‐late gestation. Timed mating analysis of embryos at different gestational ages revealed that the proportion of homozygous mutant embryos before day 11.5 was ≈25%, showing normal Mendelian inheritance, whereas embryos showed fewer homozygous mutants than expected from E12.5. And E14.5 litters did exist in homozygous mutant embryos (Figure 5C). We further examined the subcellular localization of retroviral transduced GFP‐NRAS^G12D^ and GFP‐NRAS^G12D,C181S^ in primary mouse embryonic fibroblasts (MEFs) from E13.5 embryos, which was consistent with the above‐mentioned results from BM cells and HeLa cells. (Figure S5A–C, Supporting information). The most prominent abnormalities observed in *Golga7^−/‐^* embryos were their small size, pale bodies, and accompanying hemorrhaging (Figure 5D). These findings indicate Golga7 is essential for mouse embryo development.

### 2.6 Inducible Global *Golga7* Loss does not Cause Developmental Abnormalities in Adult Mice

**Figure 6:**
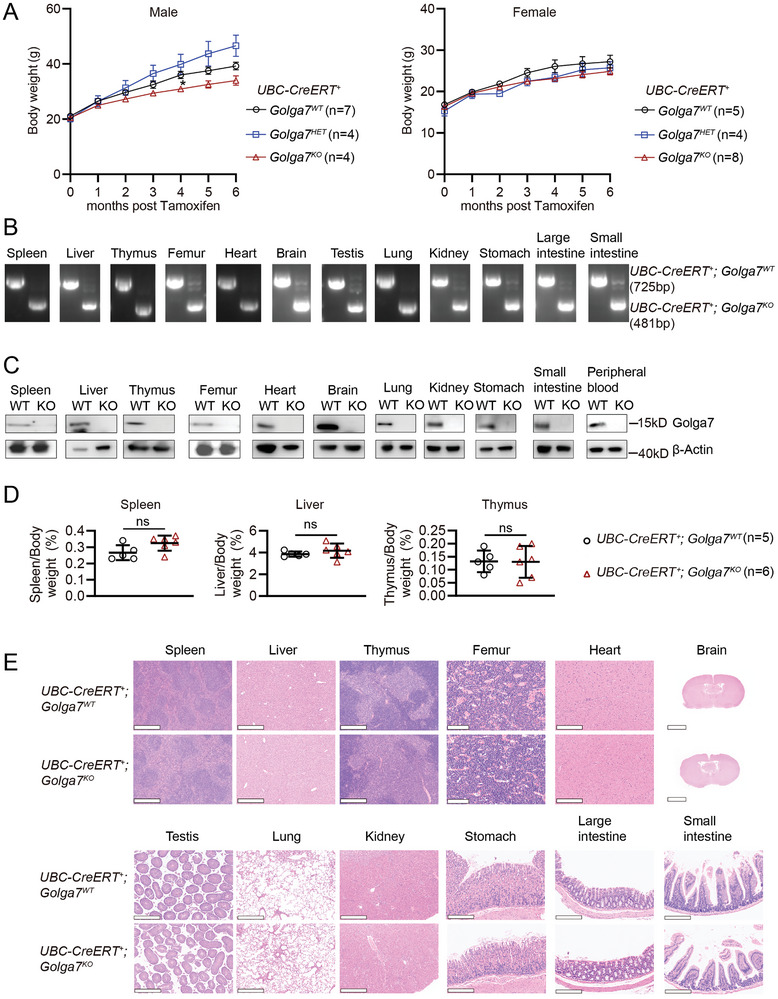
Global *Golga7* KO does not cause detectable abnormalities in adult mice. A) *Ubc‐CreERT^+^; Golga7^WT^*, *Golga7^HET^*, and *Golga7^KO^* mice were euthanized 6 months after tamoxifen treatment. Growth curve showing the body weight of mice from 0 to 6 months after tamoxifen treatment. B) PCR analysis of genomic DNA extracted from different organs 6 months after tamoxifen treatment to confirm genotypes in *Ubc‐CreERT^+^; Golga7^WT^* and *Golga7^KO^* mice. C) Western blot analysis of Golga7 expression in cells from different organs of indicated mice. D) The ratios of spleen weight, liver weight, and thymus weight to body weight in indicated cohorts. E) Representative H&E staining of organ sections. Scale bar of brain: 2.5 mm. Scale bar of thigh bone: 100 µm. Scale bar of other organs: 200 µm. Data are presented as mean ± SEM. ns, not significant.

GOLGA7 is expressed across diverse human tissues,^[^ ^33^ ^]^ a pattern similar to its ubiquitous expression in mice was observed (Figure S6A, Supporting information). To evaluate the safety of GOLGA7 as a drug target in adult mice,^[^ ^47^ ^]^ we generated an inducible global *Golga7* KO mouse line, *Ubiquitin C‐CreERT^+^; Golga7^flox/flox^* (*UBC‐CreERT^+^; Golga7^KO^*). Global *Golga7* KO was achieved by administering tamoxifen injections to one‐month‐old mice, and cohorts of *UBC‐CreERT^+^; Golga7^KO^*, *Golga7^HET^*, and *Golga7^WT^* were generated. Mice were monitored for 6 months for weight and euthanized in the end for post‐mortem analysis. No gross differences were observed in *UBC‐CreERT^+^; Golga7^KO^* or *Golga7^HET^* mice compared to *Golga7^WT^* mice. There was a trend toward lower weight in *UBC‐CreERT^+^; Golga7^KO^* male mice that were not statistically significant, and weights were more similar across the three genotypes of female mice (**Figure** **6**A). We confirmed KO efficiency in major organs at the DNA and protein levels (Figure 6B,C). Because this conditional mouse model also had *Golga7* KO in hematopoietic microenvironment cells such as mesenchymal stromal cells and endothelial cells,^[^ ^48^ ^]^ we analyzed the hematopoietic system and found no significant differences in the relative weights of spleen, liver, and thymus in *UBC‐CreERT^+^; Golga7^KO^* compared to *Golga7^WT^* mice (Figure 6D). The histopathological analysis also revealed no apparent organ structure abnormalities in *Golga7^KO^* mice compared to *Golga7^WT^* mice (Figure 6E). CBC analysis of PB was consistent with those observed in the *Mx1‐Cre* mice except that PLT levels in the *UBC‐CreERT^+^; Golga7^KO^* mice were slightly increased and T cells were slightly decreased, but both were within the normal range (Figure S6B,C, Supporting information).^[^ ^49^ ^]^ Finally, flow cytometry analysis showed no significant differences in the number of HSCs in *UBC‐CreERT^+^; Golga7^KO^* compared to *Golga7^WT^* mice (Figure S6D, Supporting information). Taken together, these findings support that there is a viable therapeutic index for GOLGA7 inhibition as a therapeutically useful target for *NRAS*‐driven tumors.

## 3 Discussion

Despite the discovery of NRAS palmitoylation more than two decades ago, the clinical relevance of this modification in treating *NRAS*‐driven cancers remains unclear. Until recently, a genome‐wide CRISPR/Cas9 screen identified *GOLGA7* as a unique synthetic lethal gene in *NRAS*‐mutant leukemia cell lines.^[^ ^32^ ^]^ Later, our study also revealed that *GOLGA7* specifically sustains the proliferation and survival of human cancer cells driven by *NRAS* mutations, but not those with *KRAS* mutations.^[^ ^31^ ^]^ In this study, we recapitulated similar results in mice, demonstrating that the loss of *Golga7* inhibited the oncogenic Nras signaling pathway within BM cells. This suppression led to decreased colony formation ability in leukemic BM cells harboring *Nras^G12D^* allele, which delayed the onset and progression of *Nras* ^*G12D*^‐driven CMML‐like MPN. Notably, despite the loss of GOLGA7 disrupting NRAS anterograde trafficking and resulting in its cis‐Golgi accumulation,^[^ ^31^ ^]^ NRAS can signal from Golgi endomembrane to some extent,^[^ ^43^ ^]^ such that all those *Nras^G12D/G12D^; Mx1‐Cre^+^; Golga7^KO^* mice ultimately developed CMML‐like MPN.

GOLGA7 has long been regarded as an auxiliary participant in the heterodimeric RAS‐PAT complex, facilitating the palmitoylation of RAS proteins across various species.^[^ ^50^, ^51^ ^]^ However, the loss of *Golga7* significantly reduces the membrane localization of GFP‐NRAS^G12D^ in primary hematopoietic cells from mice, while leaving its palmitoylation level unaltered.^[^ ^28^ ^]^ This intriguing finding is consistent with previous observations reported in human cancer cells.^[^ ^31^ ^]^ Accordingly, a recent structural biology study demonstrated, utilizing cryo‐electron microscopy, that a direct interaction exists between GOLGA7 and the ZDHHC9 subfamily of PATs.^[^ ^27^ ^]^ This study clarified that GOLGA7 does not directly participate in the catalytic process, but instead stabilizes PATs. Furthermore, GOLGA7 is not only recognized as a peripheral membrane‐associated protein primarily localized at the Golgi, but it also participates in vesicular trafficking between the Golgi and the PM when interacting with ZDHHC5.^[^ ^52^, ^53^ ^]^ Thus, these results indicate that palmitoylation is necessary but not sufficient for NRAS membrane localization.

Additionally, since several genes including ICMT and VPS35 have been reportedly involved in NRAS subcellular localization,^[^ ^54^, ^55^ ^]^ it is reasonable to speculate that GOLGA7 may also play a key role in the NRAS‐specific intracellular trafficking. It's worth mentioning that the absence of RAB27B can inhibit the PM localization of NRAS, and that it significantly impedes the development of leukemia induced by *Nras* mutations in mice.^[^ ^41^ ^]^ Unlike GOLGA7, RAB27B interacts with ZDHHC9 and modulates ZDHHC9‐mediated NRAS palmitoylation, and RAB27B KO diminishes the level of NRAS palmitoylation. Overexpression of the ZDHHC9‐GOLGA7 complex only partially rescues the phenotypes associated with RAB27B deficiency, suggesting that RAB27B may affect other enzymes catalyzing NRAS palmitoylation. Therefore, these findings suggest that Golga7 is more likely to exert its unique effect on NRAS‐specific intracellular translocation through the regulation of NRAS membrane trafficking.

Based on the significant efficacy of Golga7 loss in *Nras*‐driven leukemia, it seems that interfering with the association of GOLGA7 within RAS‐PAT complex would be a sensible strategy for targeting this non‐enzymatic protein. Non‐enzymatic proteins were considered to be non‐targetable. However, new approaches, such as proteolysis‐targeting chimera (PROTAC) technology, are turning these proteins targetable.^[^ ^56^ ^]^ We may screen and modify high affinity GOLGA7‐binding compounds to enable them to recruit proteasomes to degrade the targeted protein in the future.

However, some serious lessons should be learned in the development of inhibitors targeting RAS prenylation.^[^ ^17^ ^]^ Since farnesylation of RAS by farnesyltransferase (FTase) is a prerequisite for membrane association, extensive effort has been exerted in developing therapeutic strategies to target RAS FTase, both in academia and the pharmaceutical industry.^[^ ^38^ ^]^ Unfortunately, the therapeutic efficacy of FTase inhibitors in RAS‐driven cancer is hindered by the compensatory effect of the functional redundancy of Geranylgeranyltransferase (GGTase), another alternative prenylation enzyme. To overcome this issue, the combination of inhibitors targeting both FTase and GGTase was used to completely block the membrane association of RAS proteins.^[^ ^57^ ^]^ However, owing to the inhibition of all RAS and RHO family proteins as well as some RAB proteins, the combination therapy exhibited excessive toxicity, thereby limiting the clinical applicability of therapeutic interventions.

Therefore, we made efforts to test the safety of *GOLGA7*, which is a ubiquitous housekeeping gene that rarely undergoes mutation in human beings. Nonetheless, constitutive *Golga7*‐null is embryonically lethal, we discovered that *Golga7* KO does not result in severe hematopoietic or other organ abnormalities in adult mice. First, even if the function of endogenous wild‐type NRAS is inhibited, the functions of other RAS proteins are not affected and are sufficient for normal development.^[^ ^58^ ^]^ Several studies have revealed that distinct regulatory mechanisms dictate NRAS PM localization compared to other RAS family proteins.^[^ ^54^, ^59^ ^]^ More importantly, we found that GOLGA7 loss uniquely affects the PM localization of NRAS but does not significantly alter the PM localization of other palmitoylated RAS proteins, such as KRAS4A and HRAS.^[^ ^31^ ^]^ With the help of its KIKK motif, mono‐palmitoylated KRAS4A therefore does not depend on GOLGA7 to reach the PM, given that the palmitoylation mutant of KRAS4A did not impede leukemia development.^[^ ^60^ ^]^ For HRAS carrying two palmitoylation sites, it reportedly has two distinct routes to reach PM, which may be modified by different PATs in that process.^[^ ^54^, ^61^ ^]^ All these results suggest that GOLGA7 is a determinant of NRAS‐specific membrane trafficking with minimal off‐target effects.

Taken together, our findings uncover the crucial roles of *Golga7* in *Nras^G12D^*‐mutant CMML and normal development, and provide preclinical insights into the efficacy and safety profile for the future development of GOLGA7‐targeting interventions.

## 4 Experimental Section

The study examined male and female animals, and sex‐dimorphic effects were reported.

### Mice

The parental mouse strain had a genetic background of C57BL/6(>N10). The *Golga7* conditional knockout mouse model was designed by the laboratory and commissioned for construction and production by Shanghai Bangyao Biotechnology Co. Ltd.

### Complete Blood Count (CBC) and Histopathology

All blood samples were processed for routine blood cell analysis using the automated blood analyzer XN‐1000 V from SESAME (Sysmex Corporation). The mouse tissues were fixed in 4% paraformaldehyde and further processed for histopathology at Hubei BIOOS Biotechnology Co., Ltd.

### Statistics

The normality of the data was tested using the Shapiro–Wilk normality test. For nonparametric data, the Mann–Whitney U test was used for comparison between the two groups. Data with normal distribution were analyzed by two‐tailed *t*‐test between two groups. For multiple comparisons, data with normal distribution were analyzed by two‐way ANOVA. Data were analyzed using Prism 10.0 software.

### Study Approval

The animal care and use procedures for the mice were conducted in accordance with animal care standards, with all protocols receiving approval from the Animal Use Committee at Shanghai Jiao Tong University School of Medicine, China (IACUC Issue Number: JUMC‐2023‐046‐B).

## Conflict of Interest

The authors declare no conflict of interest.

## Authors’ contributions

B.J., L.Y., and R.Z. contributed equally to this work. R.R. and P.L. conceived and supervised the whole study. B.J., L.Y., and R.Z. performed the experiments and analyzed the data. B.J. and P.L. performed animal experiments. W.H., X.W., C.L., P.W., P.X., J.W., Z.F., D.L., Z.X., J.L., S.J., Q.Z., M.W., and S.W. provided technical or material supports, such as reagents, animal experiments, data analysis and so on. The paper was written and revised by B.J., L.Y., R.Z., P.L., and R.R.

## Supporting information



Supporting Information

## Data Availability

The RNA‐Seq data had been deposited to NCBI database under Bioproject ID PRJNA1207470. The WES data had been deposited to NCBI database under Bioproject ID PRJNA1207303.
