# Supplementary material for: Loss of Golga7 Suppresses Oncogenic Nras‐Driven Leukemogenesis without Detectable Toxicity in Adult Mice
Source: Adv Sci (Weinh). 2025 Mar 17;12(18):2412208. doi: 10.1002/advs.202412208 (PMC12079550; doi:10.1002/advs.202412208)

**Supplemental Information**

**Supplemental Materials and Methods**

**Mouse strains**

The *Golga7* gene consists of 5 exons, with 4 coding regions, and the lengths of these coding exons are all multiples of 3 nucleotides. Therefore, only the 1st exon containing the start codon ATG was designated as the flox region (111 bp). Utilizing CRISPR/Cas9 technology, sgRNAs targeting the desired gene were designed and constructed, and target sites within the intronic regions flanking the 1st exon were designed for cleavage. Simultaneously, *Cas9* mRNA was transcribed in vitro. Concurrently, donor fragments with loxP-containing homologous arms were constructed and precisely integrated via homologous recombination upstream of the 1st exon within approximately 50 bp of the non-coding region and downstream within 100-200 bp of the intron. Notably, the insertion of loxP upstream of the 1st exon may also affect the functionality of the promoter region.

Breeding was conducted using wild-type C57BL/6 mice purchased from Shanghai Slac Laboratory Animal Co., Ltd., aged 6-8 weeks. All breeding and experimental mice were maintained under SPF barrier conditions. Genotyping of mice is described in the "Genomic PCR" section.

GuideRNA information:

*Golga7*-sgRNA-L1: TGCCCGAACTCGCAGAGCGGCGG

*Golga7*-sgRNA-L2: CCGAACTCGCAGAGCGGCGGGTC

RE: GACCCGCCGCTCTGCGAGTTCGG

*Golga7*-sgRNA-R1: tcatgggaaaagatatacgaagg

*Golga7*-sgRNA-R2: aaagatatacgaaggaggtacgg

A hematopoietic specific knockout (KO) mouse strain of Golga7 gene was generated by crossing the *Golga7^flox/flox^* strain with the B6.Cg-Tg(Mx1-cre)1Cgn/J mouse strain (Stock No. 003556, Jackson Laboratory) to generate *Mx1-Cre^+^; Golga7^flox/flox^* mice*.* The genotypes are wild-type *Mx1-Cre^+^;*  *Golga7^+/+^*(*Mx1-Cre^+^; Golga7^WT^*), *Mx1-Cre^+^; Golga7 ^flox/flox^* (*Mx1-Cre^+^; Golga7^KO^*), and *Mx1-Cre^+^; Golga7 ^flox/+^* (heterozygotes; *Mx1-Cre^+^; Golga7^HET^*). Upon expression of Cre recombinase and cleavage of the loxP sites, the flox region was excised, disrupting normal Golga7 protein expression (Figure 1).

The construction of conditional *Nras ^LSL-G12D/+^* mice was consistent with previous reports and was a gift from Jing Zhang. ^1^ *Nras^LSL-G12D/+^* mice were crossed with the forementioned *Mx1-Cre* mouse strain (Stock No. 003556, Jackson Laboratory) to generate *Nras^G12D/+^;* *Mx1-Cre^+^* mice*.*

**Genomic PCR**

**Golga7 in Figure S1A:**

Forward: ATCTGACCCCGTGTCTTGCC

Reverse: TGGTCTTTCTCCAAATACTAGACCG

**Golga7 in Figure 5B:**

Forward: TCAACGTGCGGCACAGAAG

Reverse: TGTGTCGGCATCCTGTTAAG

**Mx1-Cre:**

Forward: GTGAGTTTCGTTTCTGAGCTCC

Reverse: CGGTTATTCAACTTGCACCA

**Immunofluorescence (IF) and live-cell imaging**.

*Golga7^+/+^* and *Golga7^-/-^* mouse embryonic fibroblast (MEF) cells, *Mx1-Cre^+^; Golga7^WT^* and *Mx1-Cre^+^; Golga7^KO^* bone marrow (BM) cells and HeLa cells were infected with MigR1 vector expressing *GFP* and *Nras^G12D^* (MigR1-GFP-NRAS^G12D^) or MigR1-GFP-KRAS^G12D^. After 2 days, cells were incubated with Alexa Fluor 647-conjugated wheat germ agglutinin (WGA, Thermo Fisher Scientific, catalog no. W32466) for 10 minutes at 37 ^o^C to stain the PM. Subsequently, the cells were washed with cold PBS, fixed in 4% PFA for 10 minutes, and then cell nuclei in PBS were stained with Hoechst 33342. Subsequently, cells were imaged by a confocal laser scanning microscope (Leica TCS SP8, Wetzlar, Germany) with a 63-fold oil-immersion objective lens. The captured IF images were processed by NIH ImageJ software. Pearson coefficient colocalization analysis was performed using the plug-in Coloc2 of ImageJ.

**Cell fractionation**

Cytoplasmic and plasma membrane protein fractions from 4 ×10^7^ BM cells were isolated with Minute™ plasma membrane isolation kit (Invent Biotechnologies) according to the manufacturer’s instructions.

**Acyl Resin-Assisted Capture (Acyl‑RAC) assay**

Detection of S-palmitoylated NRAS^G12D^ proteins was conducted using the commercially available CAPTUREome™ S-Palmitoylated Protein Kit (Badrilla, K010-311) according to the manufacturer’s instructions.

**Colony-forming unit (CFU) assay**

For mouse BM CFU assays，1×10^5^ whole BM cells were isolated from *Nras^G12D/G12D^; Mx1-Cre^+^;*  *Golga7^WT^* or *Nras^G12D/G12D^; Mx1-Cre^+^;*  *Golga7^KO^* mice 12 weeks post pI-pC and plated in MethoCult M3231 methylcellulose medium (StemCell Technologies). The cells were stimulated with murine GM-CSF 0.2 ng/mL (Peprotech). Colonies were identified and counted at day 12.

In addition, 2×10^4^ whole BM cells were isolated from *Mx1-Cre^+^; Golga7^WT^* or *Mx1-Cre^+^; Golga7^KO^* mice 12 weeks post pI-pC and plated in MethoCult M3434 methylcellulose medium (StemCell Technologies). Cells were kept in an incubator at 37 °C and 95% humidity in an atmosphere of 5% CO_2_. Colonies were identified and counted at day 7.

**Flow cytometric analysis of hematopoietic tissues**

All antibodies for flow cytometry were purchased from Biolegend (San Diego, CA) and BD Biosciences (San Jose, CA). For lineage analysis of peripheral blood (PB), BM, and spleen (SP) cells, flow cytometric analyses were performed as previously described.^2, 3^ In brief, cells were lysed with red blood cell lysis buffer and fluorochrome-conjugated antibodies against CD11b, CD19 , CD3𝜖, CD45, and Gr-1 were used for staining. hematopoietic stem cells (HSCs) were stained with fluorochrome-labeled antibodies against Sca-1, c-kit, CD34, CD16/32, CD127, CD150, CD48, and CD135. All of the antibodies were listed in Table S1.Data were analyzed with FlowJo software (Tree Star).

**Signaling studies**

For whole BM cells, cells were cultured in RPMI-1640 medium plus 0.5% BSA for 0.5 hours, then cell pellets were immediately collected by centrifugation at 16,000*g* for 15 seconds and snap frozen. Cells were lysed using 1× sodium dodecyl sulfate (SDS) sample loading buffer. Protein were then separated by electrophoresis on a polyacrylamide gel, transferred to a nitrocellulose membrane, and probed with appropriate primary and secondary antibodies. The luminescent signals on the membrane were detected using the LumiQ HRP Substrate Solution kit (share-bio, SB-WB012), and the blots were visualized with the ChemiDoc MP imaging system (Bio-Rad)

**RNA-Seq analysis of mouse BM cells**

Total RNA was isolated from 5 ×10^6^ BM cells from *Nras^G12D/G12D^; Mx1-Cre^+^* *Golga7^WT^;* (*n* = 2), and *Nras^G12D/G12D^; Mx1-Cre^+^; Golga7^KO^* (*n* = 2) mice 8 weeks after polyinosinic-polycytidylic (pI-pC) induction and from age-matched normal control (NC) mice (*n* = 2). RNA library preparation was performed as described in the Illumina TruSeq Stranded Total RNA LT sample preparation kit with RiboZero Gold (Illumina Inc). RNA-Seq was performed using an Illumina Nova 6000 platform. Demultiplexed raw sequencing data were cleaned using fastp (v0.20.1), and the clean reads were aligned to the mouse genome (mm10) using STAR (v2.7.10a) with Ensemble annotation (GRCm38.93). Gene-level expression values were calculated using StringTie (v2.1.7). Data normalization and differential expression analysis were performed using DESeq2 (v1.28.1). Gene Ontology (GO) enrichment analysis was performed using clusterProfiler (v3.16.1).

**Whole exome sequencing (WES) of mouse leukemia cells and data analysis**

Genomic DNA was extracted from BM cells and SP tissues of moribund *Nras^G12D/G12D^; Mx1-Cre^+^;*  *Golga7^KO^* (*n* = 5) and matched NC (*n*=3) mice (Table S2) using a Genomic DNA Purification Kit (Promega). Exome sequences were enriched from 0.4 μg genomic DNA using the Agilent SureSelectXT Mouse All Exon library (Agilent, USA, Catalog #: 5190-4643) according to the manufacturer’s instructions. Captured libraries were enriched by PCR to add index tags in preparation for sequencing. The samples were then purified using the AMPure XP system (Beckman Coulter, Beverly, USA). The Agilent 5400 system (AATI) (Agilent, USA) was used to assess the size distribution of the libraries. Clean data were aligned to the reference genome using Burrows Wheeler Aligner (BWA) software and Samblaster to generate the BAM files.^4, 5^ Next, Sambamba was used to sort the BAM files and flag duplicate reads based on chromosome position.^6^ Germline single-nucleotide polymorphism (SNP) and insertion-deletion (InDel) calling was performed using SAMtools,^7^ and variants were annotated using Annovar.^8^ The filter parameters for SNPs and InDels were follows: QUAL ≥ 20; DP ≥ 4; MQ ≥ 30. For further analysis, we created a union of SNPs/InDels from the NC group and used this list to filter the KO group, identifying only the unique variants specific to the KO group.

**Supplemental References**

1. Wang, J.; Liu, Y.; Li, Z.; Wang, Z.; Tan, L. X.; Ryu, M.-J.; Meline, B.; Du, J.; Young, K. H.; Ranheim, E.; Chang, Q.; Zhang, J., Endogenous oncogenic Nras mutation initiates hematopoietic malignancies in a dose- and cell type-dependent manner. *Blood* **2011,** *118* (2), 368-379.

2. Liu, P.; Jiao, B.; Zhang, R.; Zhao, H.; Zhang, C.; Wu, M.; Li, D.; Zhao, X.; Qiu, Q.; Li, J.; Ren, R., Palmitoylacyltransferase Zdhhc9 inactivation mitigates leukemogenic potential of oncogenic Nras. *Leukemia* **2016,** *30* (5), 1225-8.

3. Li, D. H.; Zhang, Y. Y.; Qiu, Q. S.; Wang, J. Z.; Zhao, X. M.; Jiao, B.; Zhang, X. L.; Yu, S. H.; Xu, P. F.; Dan, Y. Q.; Xiao, X. H.; Wang, P. H.; Liu, M. Z.; Xia, Z. Z.; Huang, Z. S.; Zhang, R. H.; Li, J. Y.; Xie, X.; Zhang, Y.; Liu, C. X.; Liu, P.; Ren, R. B., IRF8 Impacts Self-Renewal of Hematopoietic Stem Cells by Regulating TLR9 Signaling Pathway of Innate Immune Cells. *ADVANCED SCIENCE*.

4. Li, H.; Durbin, R., Fast and accurate long-read alignment with Burrows-Wheeler transform. *Bioinformatics* **2010,** *26* (5), 589-95.

5. Faust, G. G.; Hall, I. M., SAMBLASTER: fast duplicate marking and structural variant read extraction. *Bioinformatics* **2014,** *30* (17), 2503-5.

6. Tarasov, A.; Vilella, A. J.; Cuppen, E.; Nijman, I. J.; Prins, P., Sambamba: fast processing of NGS alignment formats. *Bioinformatics* **2015,** *31* (12), 2032-4.

7. Li, H.; Handsaker, B.; Wysoker, A.; Fennell, T.; Ruan, J.; Homer, N.; Marth, G.; Abecasis, G.; Durbin, R., The Sequence Alignment/Map format and SAMtools. *Bioinformatics* **2009,** *25* (16), 2078-9.

8. Wang, K.; Li, M.; Hakonarson, H., ANNOVAR: functional annotation of genetic variants from high-throughput sequencing data. *Nucleic Acids Res* **2010,** *38* (16), e164.

**Supplemental Figure Legends**

**Figure S1. *Golga7* deficiency reduces *Nras^G12D^*-induced leukemic burden.**

1. Representative PCR analysis of genomic DNA to detect WT allele, LSL allele, and recombined LSL allele (1 LoxP allele) of NRAS, and to confirm genotypes of *Mx1-Cre^+^; Golga7^WT^, Golga7^HET^*, and *Golga7^KO^* mice.
2. Frequencies of myeloid cells, T lymphocytes, and B lymphocytes in PB were monitored before and every 4 weeks after pI-pC injections. *P*-values were calculated by two-way ANOVA ( *Nras^G12D/G12D^; Mx1-Cre^+^;*  *Golga7^WT^* vs *Nras^G12D/G12D^; Mx1-Cre^+^;*  *Golga7^KO^*).
3. Representative H&E staining of spleen and liver sections at 16 weeks of indicated mice after pI-pC injections. Scale bar: 500 μm.
4. The percentage of myeloid cells, T lymphocytes, and B lymphocytes among CD45^+^ cells from spleen (SP) of indicated mice. The P-value was calculated by unpaired Student’s *t*-test.
5. The percentage of myeloid cells, T lymphocytes, and B lymphocytes among CD45^+^ cells from BM cells of indicated mice. The P-value was calculated by unpaired Student’s *t*-test.
6. Flow cytometric analysis of BM cells.
7. Quantitative analysis of the LK, CMP, GMP, MEP compartment in BM. The P-value was calculated by unpaired Student’s *t*-test.
8. The percentage of LT-HSCs and ST-HSCs in BM. Results are presented as mean ± SEM. The P-value was calculated by unpaired Student’s *t*-test.
9. 1×10^5^ whole BM cells isolated from *Nras^G12D/ G12D^; Golga7^WT^; Mx1-Cre^+^*, *Nras^G12D/ G12D^; Golga7^HET^; Mx1-Cre^+^*, and *Nras^G12D/ G12D^; Golga7^KO^; Mx1-Cre^+^* mice were plated in MethoCult M3231 methylcellulose medium without cytokine or with 0.2 ng/ml mGM-CSF. Colonies were counted on day 12 of culturing. Data are presented as the mean ± SEM. Statistical comparisons were performed by unpaired Student’s *t*-test and Mann-Whitney test in Prism. *Golga7^HET^* without mGM-CSF (left graph), ns *P*=0.9885. *Golga7^HET^* with mGM-CSF (right graph), ns , not significant, *P*=0.5835, **P*<0.05; ***P*<0.01; ****P*<0.001; *****P*<0.0001.

**Figure S2. GOLGA7 knockout does not modulate homeostasis of NRAS.**

1. Levels of Nras^G12D^ and Golga7 protein in BM whole-cell lysates (WCL), cytoplasmic fraction (Cyto.), and plasma membrane fraction (Mem.) from a representative western blot (n = 3). GAPDH (glyceraldehyde-3-phosphate dehydrogenase) and Na, K-ATPase were used as cytoplasm and plasma membrane markers, respectively.
2. Quantification of data presented in (**A**). Data are presented as mean ± SD. Statistical comparisons were performed by unpaired Student’s *t*-test in Prism.
3. HeLa cells were transfected with scramble single guide RNA (sgRNA, SCR)or sgRNA against GOLGA7(sgGOLGA7), respectively. HeLa cells ± sgGOLGA7 overexpressing GFP-NRAS^G12D^ was incubated with cycloheximide (CHX) and analyzed by western blot at the indicated time points. CHX that inhibits nascent protein synthesis was added at 50 μg/mL.
4. Quantification of data presented in (**C**). Statistical comparisons were performed by two-way ANOVA in Prism. ns, not significant.
5. WBC counts levels in PB were monitored 8 weeks after pI-pC injection. *P*-values were calculated by unpaired Student’s *t*-test in Prism.
6. Photograph of spleens from mice of indicated groups 8 weeks after pI-pC injection.
7. The ratio of spleen weight: body weight of indicated mice 8 weeks after pI-pC injection. *P*-values were calculated by unpaired Student’s *t*-test.
8. The percentage of myeloid cells, T lymphocytes, and B lymphocytes among CD45^+^ cells from PB of indicated mice. The P-value was calculated by unpaired Student’s *t*-test.
9. The percentage of myeloid cells, T lymphocytes, and B lymphocytes among CD45^+^ cells from SP of indicated mice. The P-value was calculated by unpaired Student’s *t*-test.
10. The percentage of myeloid cells, T lymphocytes, and B lymphocytes among CD45^+^ cells from BM of indicated mice. The P-value was calculated by unpaired Student’s *t*-test.
11. Heatmap of differentially expressed genes (DEGs) in *Nras^G12D/G12D^; Mx1-Cre^+^;*  *Golga7^WT^* , *Nras^G12D/G12D^; Mx1-Cre^+^;*  *Golga7^KO^* and normal control (NC) bone marrow cells 8 weeks after pI-pC injection. (fold change ≥2 and false discovery rate (FDR) <0.05).
12. Venn diagrams of upregulated DEGs among *Nras^G12D/G12D^; Mx1-Cre^+^;*  *Golga7^WT^*, and *Nras^G12D/G12D^; Mx1-Cre^+^;*  *Golga7^KO^* BM cells. The representative biological processes are shown with numbers of genes in each category and corresponding FDR in parentheses. ns, not significant, **P*<0.05; ***P*<0.01; ****P*<0.001; *****P*<0.0001.

**Figure S3. *Nras^G12D/ G12D^; Mx1-Cre^+^; Golga7^KO^* mice develop CMML-like MPN after a prolonged latency.**

1. Representative flow cytometry analysis of BM and spleen (SP) cells from moribund  *Nras^G12D/ G12D^; Mx1-Cre^+^;*  *Golga7^WT^* (16 weeks after pI-pC injections), *Nras^G12D/ G12D^; Mx1-Cre^+^;*  *Golga7^KO^* (40 weeks after pI-pC injections) mice and NC (15 weeks after pI-pC injections) mice.
2. Genomic DNA was extracted from BM cells and spleen of moribund *Nras^G12D/ G12D^; Mx1-Cre^+^;*  *Golga7^KO^* (*n* = 5) and matched NC mice (*n* = 3) for whole exome sequencing.

**Figure S4. *Golga7* loss has minimal impact on normal hematopoiesis in adult mice.**

1. Analysis of different hematopoietic stem progenitor cell populations in the total BM of *Mx1-Cre^+^;*  *Golga7^WT^* (*n* = 6),  *Mx1-Cre^+^;*  *Golga7^HET^*(*n* = 5), and *Mx1-Cre^+^;*  *Golga7^KO^* (*n* = 6) mice. Data are presented as mean ± SEM. Statistical comparisons were performed by unpaired Student’s *t*-test in Prism. **P*<0.05
2. 2×10^4^ whole BM cells from indicated mice were plated in MethoCult M3434 methylcellulose medium. Colonies were counted on day 7 of culturing. Scale bar: 200μm.
3. Quantification of data presented in (**B**). Data are presented as the mean ± SEM. Statistical comparisons were performed by unpaired Student’s *t*-test and Mann-Whitney test in Prism. ns, not significant.

**Figure S5. Loss of *Golga7* disrupts oncogenic Nras^G12D^ PM localization in MEF cells.**

1. MEF cells from *Golga7^+/+^*, *Golga7^+/-^*, *Golga7^-/-^* mice were transduced to stably express GFP-NRAS^G12D^ by retroviral infection. A line was drawn across confocal images of cells and the signals for GFP (green), wheat germ agglutinin (WGA; red) and nuclei (Hoechst33342; blue) along the line are plotted. The original magnification was ×630. Scale bar: 25 μm.
2. MEF cells from *Golga7^+/+^* mice were transduced to stably express GFP-NRAS^G12D, C181S^, or GFP by retroviral infection. A line was drawn across confocal images of cells and the signals for GFP (green) and WGA (red) and nuclei (blue) along the line are plotted. The original magnification was ×630. Scale bar: 25 μm.
3. Pearson’s coefficient of GFP-NRAS^G12D^ and WGA signals (mean ± SEM) of data shown in (**B**). Values are the mean ± SEM from *n* = 20 cells per group. Statistical comparisons were performed by unpaired Student’s *t*-test in Prism. *****P*< 0.0001; *Golga7^+/-^*, ns, *P*= 0.1932.

**Figure S6. Global *Golga7* KO does not cause developmental abnormalities in adult mice**

1. Illustration of the expression of human and murine *GOLGA7* homologs, based on transcriptome datasets derived from the National Center for Biotechnology Information (NCBI) database.
2. The WBC counts, RBC counts, hemoglobin levels, hematocrit levels, and PLT counts from PB were monitored in indicated mice. Statistical comparisons were performed by unpaired Student’s *t*-test in Prism.
3. The percentage of myeloid cells, T lymphocytes, and B lymphocytes in CD45^+^ cells from PB. Statistical comparisons were performed by unpaired Student’s *t*-test in Prism.
4. The percentage of different hematopoietic stem progenitor cell populations in the total BM. Values are presented as the mean± SEM. Statistical comparisons were performed by unpaired Student’s *t*-test in Prism.

**Table S1. Antibody details are as follows:**

**List of antibodies used in this study.**

| **Antibody** | **Vendor** | **Catalogue Number** |
| --- | --- | --- |
| Na^+^K^+^ATPase | Abcam | ab76020 |
| GAPDH | Proteintech | HRP-60004 |
| GOLGA7 | ABclonal | Customer Antibody  Project number: WG-04524 |
| ERK | Cell Signaling Technology | 4695 |
| Phospho-ERK | Cell Signaling Technology | 4370 |
| AKT | Cell Signaling Technology | 4691 |
| Phospho-AKT (Ser473) | Cell Signaling Technology | 4060 |
| S6 | Cell Signaling Technology | 2317 |
| Phospho-S6 | Cell Signaling Technology | 4858 |
| α-Tubulin | Proteintech | HRP-66031 |
| β-Actin | Proteintech | HRP-60008 |
| Anti-rabbit HRP | Cell Signaling Technology | 7074 |
| Goat anti-Rabbit Alexa Fluor™ 555 | Abcam | ab150078 |
| Hoechst 33342 | Beyotime | C1025 |
| Biotin anti-mouse Ly-6G/Ly-6C (Gr-1) Antibody | BioLegend | 108404 |
| Biotin anti-mouse/human CD11b Antibody | BioLegend | 101204 |
| Biotin anti-mouse CD3ε Antibody | BioLegend | 100304 |
| Biotin anti-mouse/human CD45R/B220 Antibody | BioLegend | 103204 |
| Biotin anti-mouse TER-119/Erythroid Cells Antibody | BioLegend | 116204 |
| FITC Streptavidin | BioLegend | 405202 |
| CD117 (c-Kit) Monoclonal Antibody | Invitrogen | 63-1171-82 |
| Ly-6A/E (Sca-1) Monoclonal Antibody (D7), PE-Cyanine7 | Invitrogen | 25-5981-82 |
| Brilliant Violet 421™ anti-mouse CD34 Antibody | BioLegend | 152208 |
| APC anti-mouse CD16/32 Antibody | BioLegend | 101326 |
| Brilliant Violet 785™ anti-mouse CD127 (IL-7Rα) Antibody | BioLegend | 135037 |
| PE anti-mouse CD150 (SLAM) Antibody | BioLegend | 115904 |
| APC/Cyanine7 CD48 Antibody | BioLegend | 103432 |
| CD135 (Flt3) Antibody (A2F10), PE-Cyanine5 | Invitrogen | 15-1351-81 |
| FITC anti-mouse CD3ε Antibody | BioLegend | 100306 |
| APC anti-mouse CD19 Antibody | BioLegend | 115512 |
| FITC anti-mouse/human CD11b Antibody | BioLegend | 101206 |
| PE anti-mouse Ly-6G/Ly-6C (Gr-1) Antibody | BioLegend | 108408 |
| Brilliant Violet 421™ anti-mouse CD45 Antibody | BioLegend | 103134 |
| FITC anti-mouse/human CD11b Antibody | BioLegend | 101206 |
| PE/Cyanine7 anti-mouse Ly-6G Antibody | BioLegend | 127618 |

**Table S2. Mice samples used in WES.**

| Sample | Genotype | Days post pI-pC | Source of leukemia cells |
| --- | --- | --- | --- |
| 277 | *Nras^G12D/G12D^; Golga7^KO^* | 648 | Spleen |
| 278 | *Nras^G12D/G12D^; Golga7^KO^* | 235 | Spleen |
| 542 | *Nras^G12D/G12D^; Golga7^KO^* | 227 | Bone marrow |
| 549 | *Nras^G12D/G12D^; Golga7^KO^* | 227 | Bone marrow |
| 558 | *Nras^G12D/G12D^; Golga7^KO^* | 227 | Bone marrow |

**Table S3. Uncommon technical abbreviations and acronyms for specialized terms：**

RAS: Rat sarcoma

MAPK: Mitogen-Activated Protein Kinase

Raf: Raf protein kinase

MEK: Mitogen-Activated Protein Kinase Kinase

ERK: Extracellular Signal-Regulated Kinase

PI3K: Phosphatidylinositol 3-Kinase

AKT: Protein Kinase B

mTOR: mammalian target of rapamycin

ICMT: Isoprenylcysteine carboxyl methyltransferase

VPS35: Vacuolar Protein Sorting 35

RAB27B: RAB family member 27B

LoxP: Locus of X-over P1

Cre: Causes recombination

LSL: LoxP-Stop-LoxP，STOP means stop codon of a protein

Mx1: Myxovirus resistance 1

UBC: Ubiquitin Conjugating Enzyme

EIIa: adenovirus EIIa promoter

pI-pC: polyinosinic-polycytidylic acid

CHX: Cycloheximide

sgRNA: single guide RNA

SCR: scrambled sgRNA


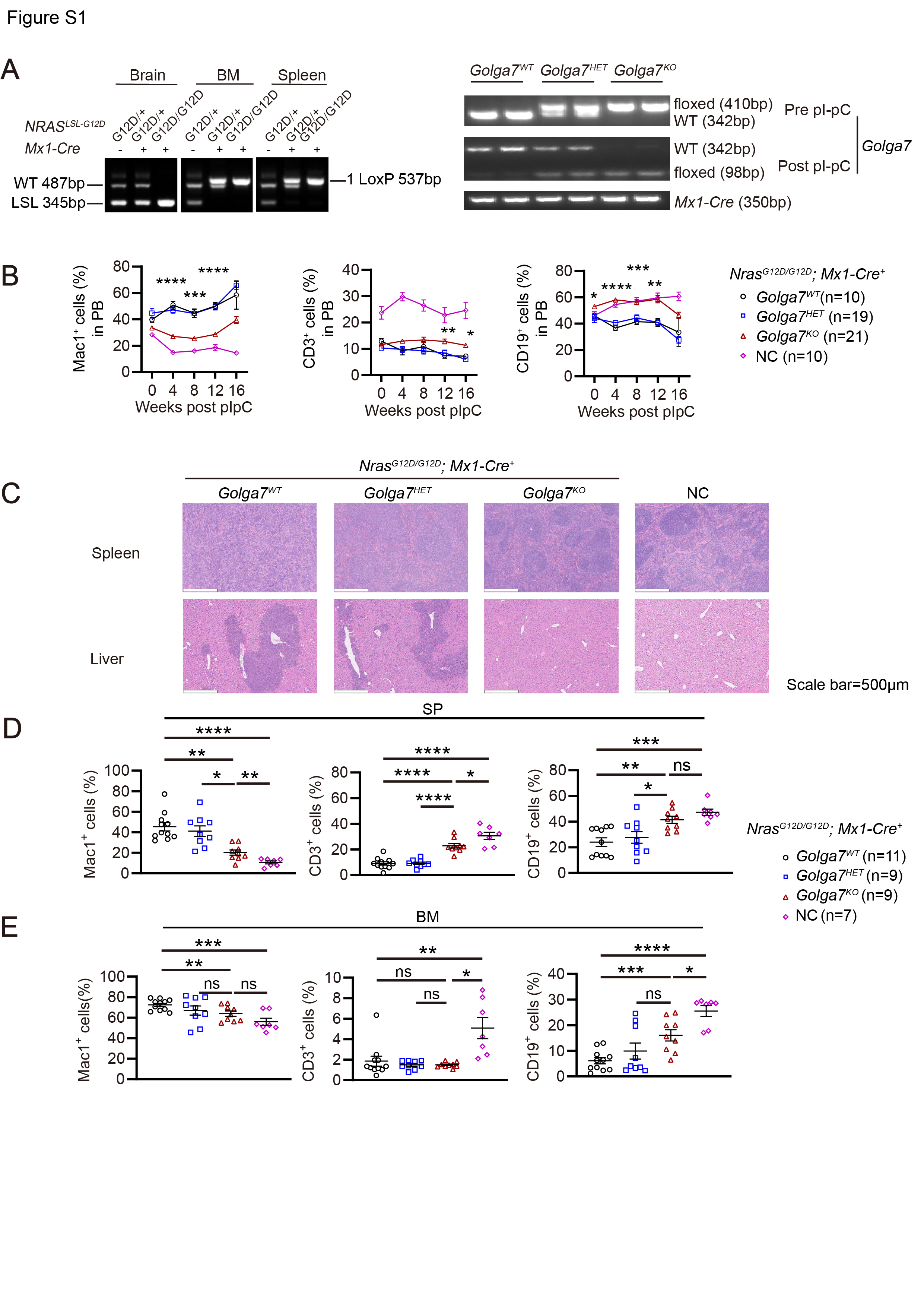

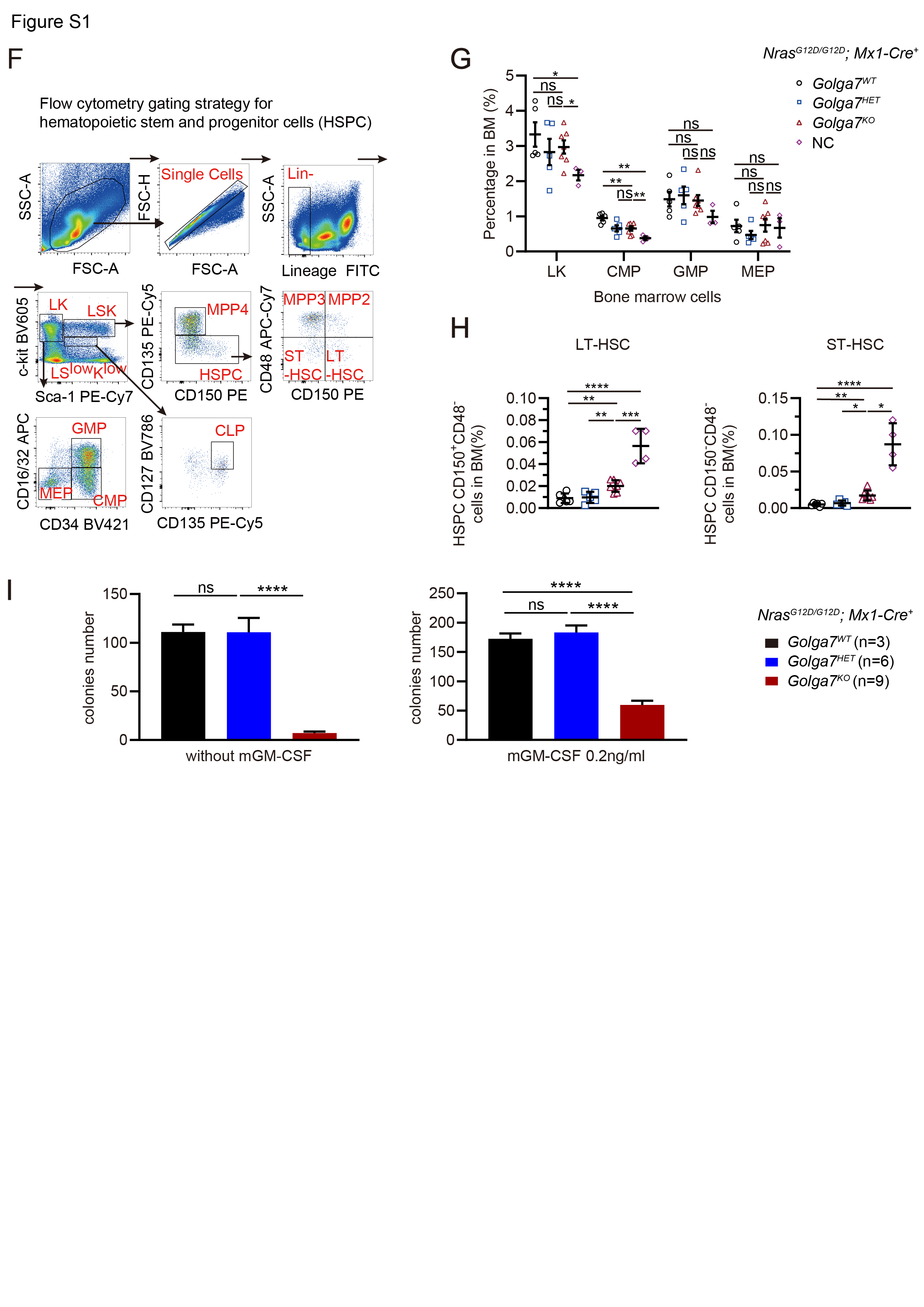

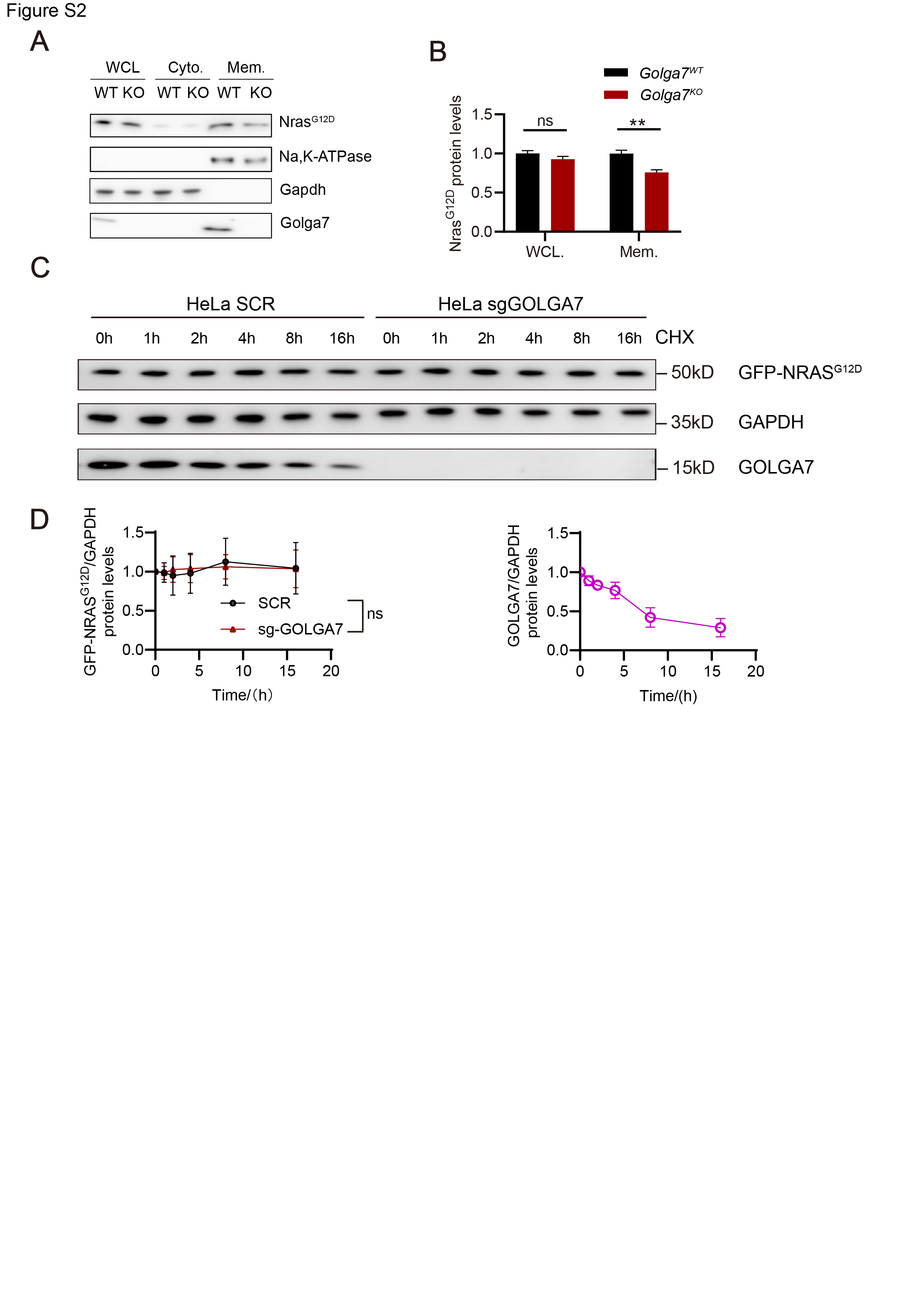

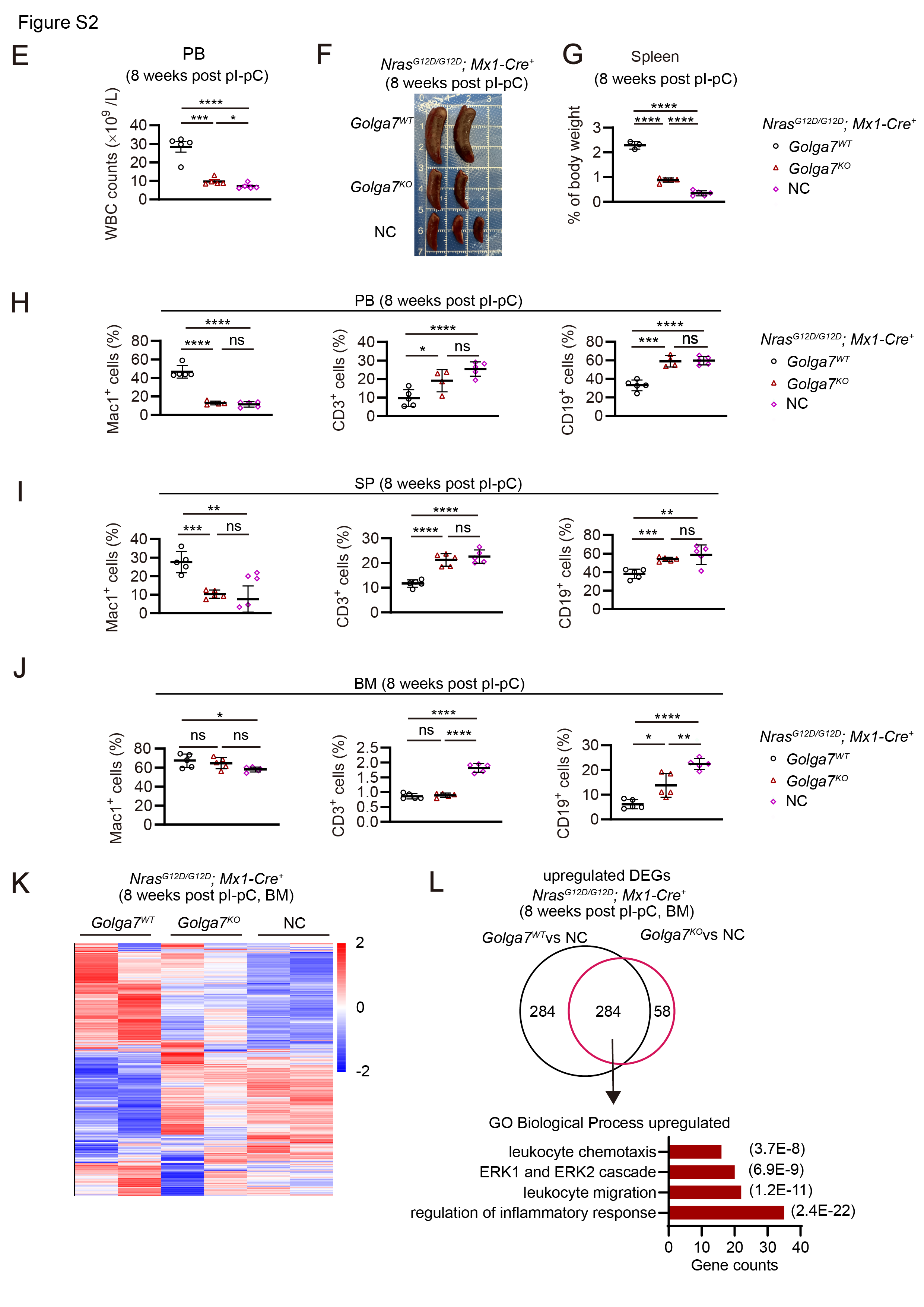

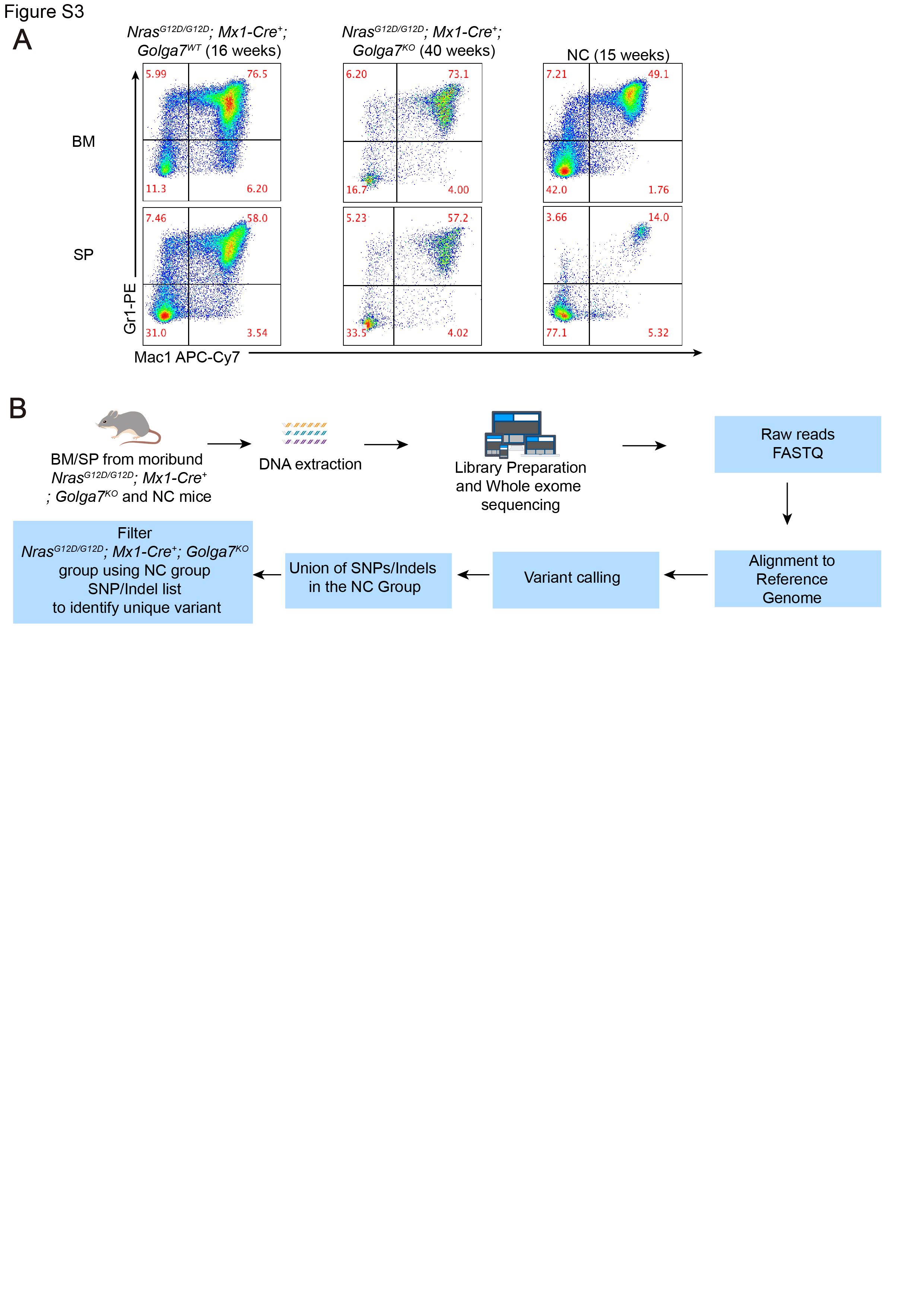

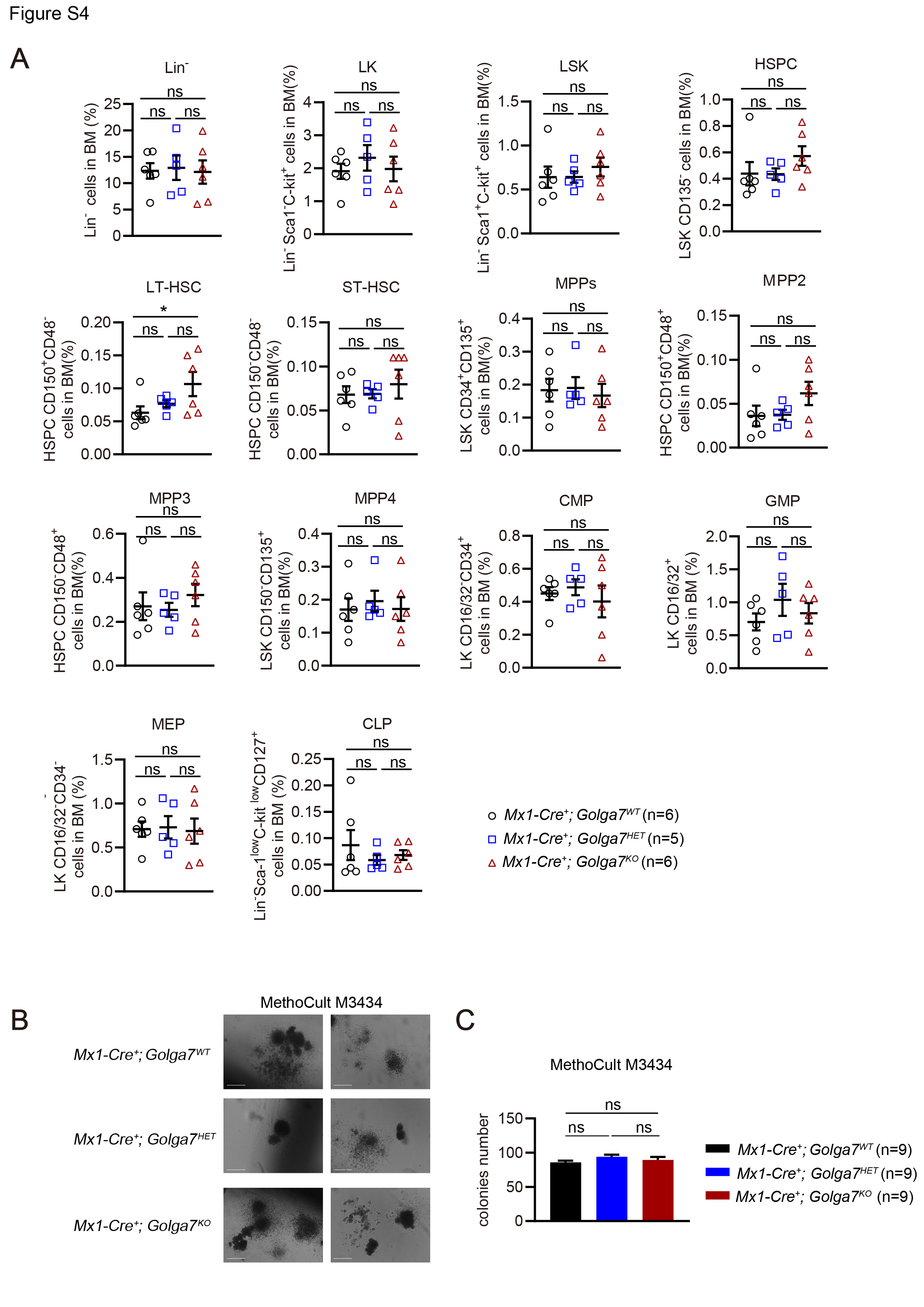

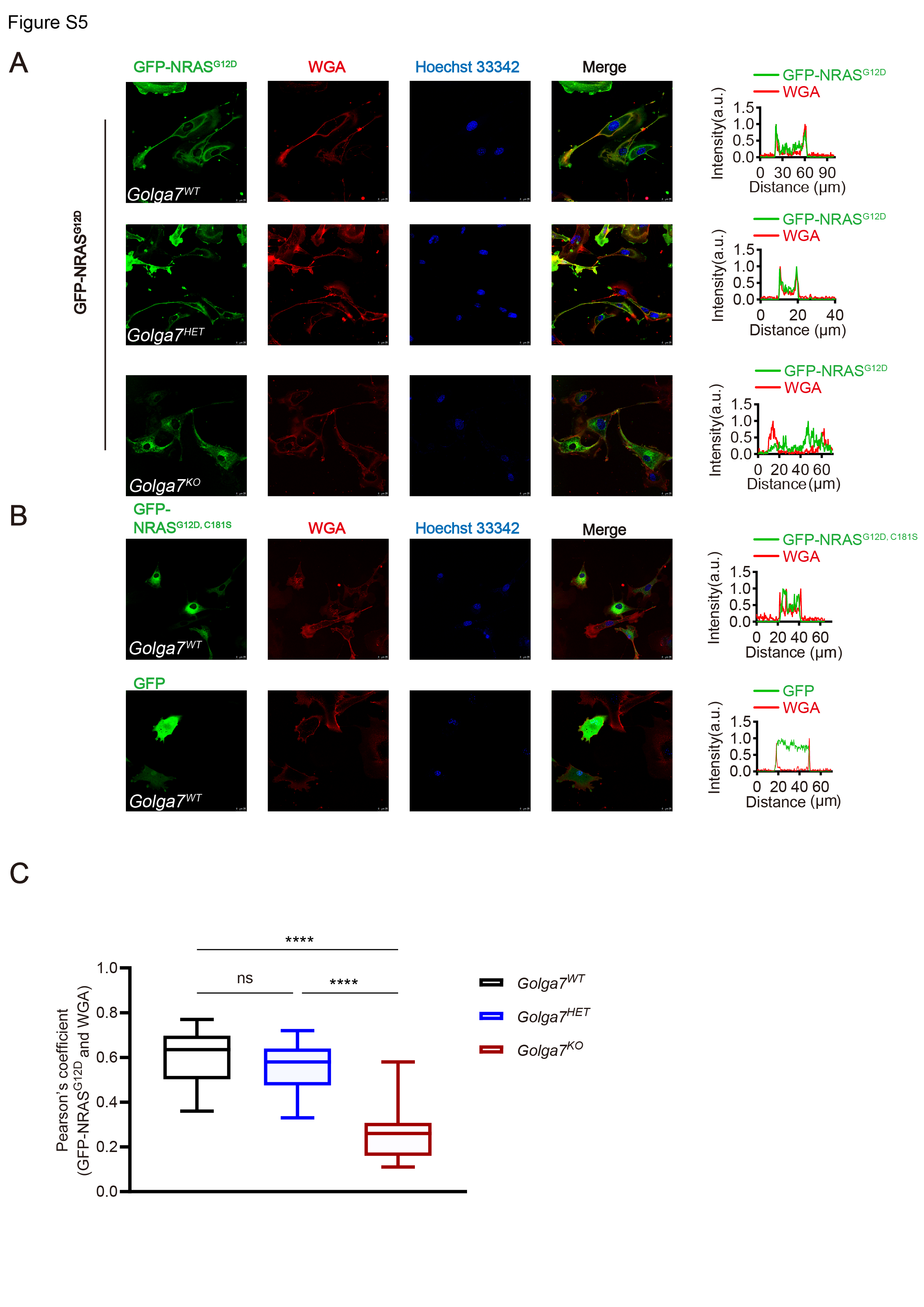

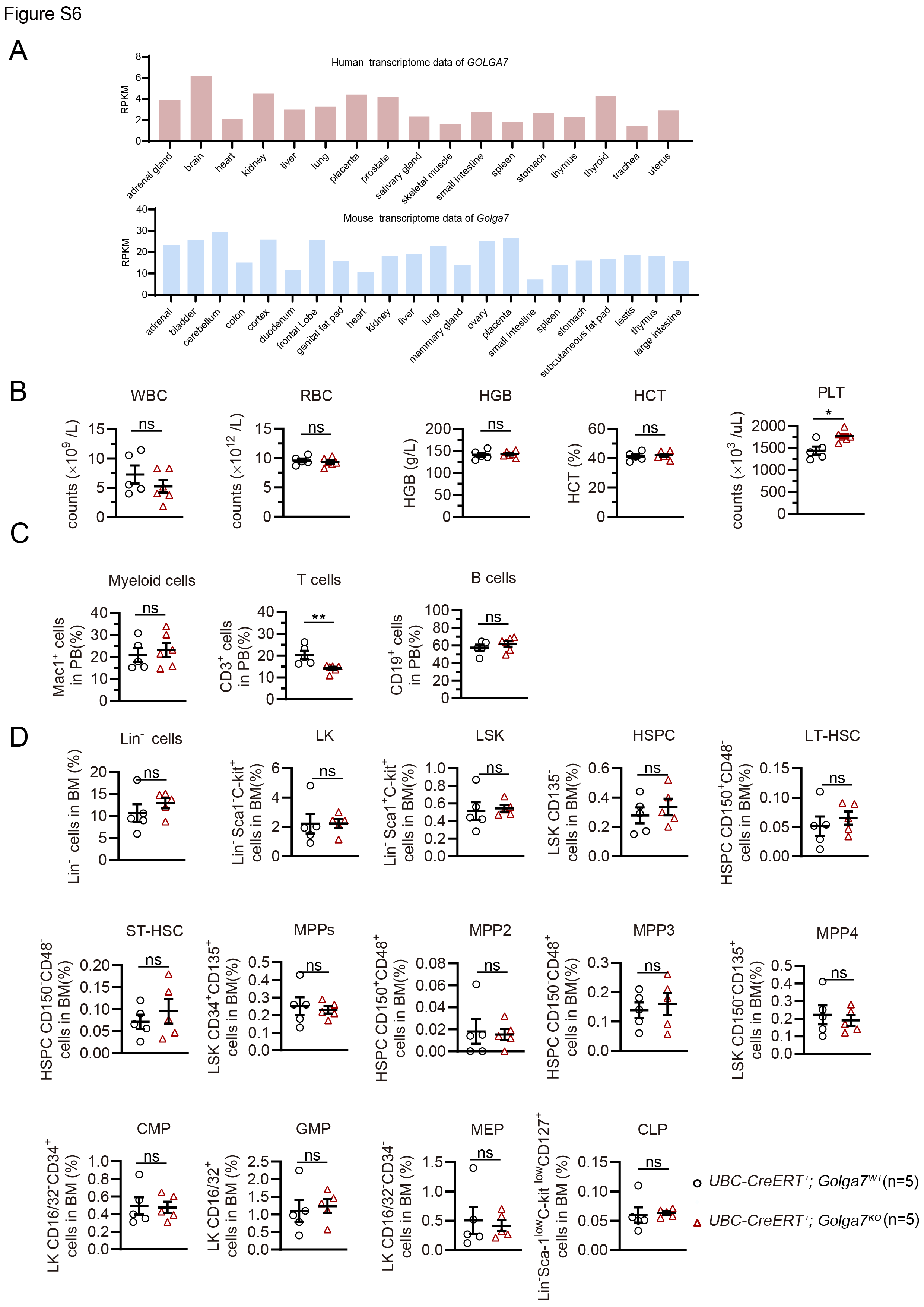

Supplement: Supplementary file 1 — Supporting Information [file ADVS-12-2412208-s001.docx]
